# Coupling of Engineered High Entropy Alloys with Semiconducting TiO_2_ Nanofilms for Scalable and Ultrafast Alkaline Hydrogen Evolution Reaction

**DOI:** 10.1002/advs.202514558

**Published:** 2025-10-15

**Authors:** Zichu Zhao, Yanzhang Zhao, Wen‐Qiang Wang, Xiaying Xin, Yan Jiao, Andrew D. Abell, Cheryl Suwen Law, Abel Santos

**Affiliations:** ^1^ School of Chemical Engineering The University of Adelaide Adelaide South Australia 5005 Australia; ^2^ Beaty Water Research Centre Department of Civil Engineering Queen's University Kingston Ontario K7L3N6 Canada; ^3^ Institute for Photonics and Advanced Sensing (IPAS) The University of Adelaide Adelaide South Australia 5005 Australia; ^4^ Department of Chemistry The University of Adelaide Adelaide South Australia 5005 Australia

**Keywords:** high entropy alloys, hydrogen evolution reaction, photoelectrocatalysis, titanium dioxide films

## Abstract

High entropy alloys (HEAs) are a promising class of electrocatalysts because of their high reactivity. However, the development of scalable synthesis strategies and fundamental understanding of their interfacial synergy with metal oxides remains underexplored. Herein, a new approach is reported for the fabrication of hybrid photoelectrocatalysts combining PtFeCoNiCu HEA structures with titanium dioxide (TiO_2_) nanofilms via sequential anodization and electrodeposition. The TiO_2_ nanofilms function as both a photoactive semiconducting framework and nanostructured substrate, enabling controlled nucleation and growth of HEA nanoparticles through a Volmer–Weber mechanism. The resulting hybrid photoelectrocatalysts exhibit outstanding hydrogen evolution reaction (HER) performance, achieving an ultralow overpotential of –11 mV at 10 mA cm^−2^ under simultaneous illumination and elevated electrolyte temperature. Mechanistic studies combining in situ Raman spectroscopy and density functional theory simulations reveal that HER occurs through three distinct stages, during which the TiO_2_ support undergoes dynamic structural and electronic evolution – from a passive scaffold to an electron‐buffering layer. This process involves Ti⁴⁺ reduction, hydrogen intercalation, and accelerated turnover of OH^*^ intermediates, which collectively enhance interfacial charge transfer and broaden active‐site availability. These findings provide new insights into the dynamic interplay between HEAs–semiconducting metal oxide substrates, enabling a generalizable design strategy for scalable, high‐performance photoelectrocatalysts.

## Graphical abstract

A facile and scalable strategy is presented to fabricate high‐performance hybrid photoelectrocatalysts combining PtFeCoNiCu HEA and TiO_2_–NFs via sequential anodization and electrodeposition, achieving an ultralow overpotential of –11 mV at η_10_ and excellent thermal stability.

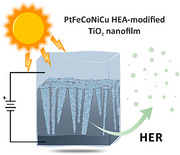

## 1 Introduction

The urgent pursuit of carbon neutrality requires dramatic advances in alternative forms of energy that are accessible, affordable, sustainable, and reliable. Electrocatalysts are promising platforms to tackle carbon dioxide emissions and address the rapidly increasing global demand for renewable energy because they can drive a broad range of reactions in an environmentally friendly manner. HEAs materials are defined by the number of principal elements (five or more), element fractions (5–35%), mixing entropy (≥ 1.5 R), and number of phases (one or two).^[^ ^1^, ^2^ ^]^ HEAs have garnered considerable attention in recent years as promising materials for catalysis.^[^ ^3^, ^4^, ^5^, ^6^ ^]^ The unique “cocktail effect” of HEAs is attributed to the synergy between different constituent metal atoms enables effective modulation of the adsorption and desorption energies of reaction intermediates and provides a high density of active sites with electron transferability.^[^ ^7^ ^]^ Consequently, electrocatalysts based on HEAs have been extensively explored in reactions such as hydrogen evolution reaction (HER),^[^ ^8^ ^]^ oxygen evolution reaction (OER),^[^ ^9^ ^]^ and carbon dioxide reduction reaction (CO_2_RR).^[^ ^10^ ^]^ Despite their potential, the synthesis of HEAs remains a critical bottleneck to precisely controlling their composition and addressing key fundamental questions about the correlation between material properties and reaction performance.^[^ ^11^ ^]^ HEAs can be fabricated by several methods, including carbothermal shock synthesis,^[^ ^12^ ^]^ rapid moving‐bed pyrolysis,^[^ ^13^ ^]^ laser ablation,^[^ ^14^ ^]^ and pulsed laser deposition.^[^ ^15^ ^]^ However, these techniques have limitations to control the composition of HEAs and require high‐cost equipment and sophisticated processes. For example, carbothermal shock synthesis and rapid moving‐bed pyrolysis require high temperatures (i.e., ≈2000 and ≈1000 K, respectively), and fast heating rates of 200 K s^−1^. These conditions impose safety risks and require complex, expensive equipment. Similarly, laser‐based synthesis methods necessitate specialist equipment and controlled safety environments to prevent harm from laser exposure. Further to that, these methods suffer from intrinsic constraints for scalability, limiting the production of HEAs to small batches. In contrast, electrodeposition offers a simple, rapid, cost‐effective, scalable, and versatile approach to control the composition, size, and geometry of HEAs through electrodeposition parameters such as time, voltage, and electrolyte composition without the need for capping or reducing agents.^[^ ^16^ ^]^ The electrodeposition process relies on a conductive substrate to adsorb free metal ions from the electrolyte, which are deposited under the action of an externally applied electric field. As such, this fabrication approach also opens new avenues to combine HEAs with a broad range of substrates and materials. A potential path is the combination of HEAs with semiconductors to further enhance the performance of this platform material through photoelectrocatalysis (PEC).^[^ ^17^ ^]^ In this system, HEAs can efficiently capture electrons generated by the semiconductor under illumination to reduce electron–hole recombination in the semiconductor and enable an efficient utilization of electrons to drive reactions. Of all semiconductors, anodized titanium dioxide (TiO_2_) – benchmark photocatalysts – in the form of films, nanotubes, and nanoflakes, has been demonstrated as an excellent supporting material to develop PEC systems.^[^ ^18^, ^19^, ^20^ ^]^ TiO_2_ is also a n–type semiconductor catalyst, which favours cathodic reactions such as HER for generating green hydrogen gas.^[^ ^21^ ^]^ So far, the combination of TiO_2_ structures with metal atoms for PEC‐driven HER has been limited to single‐atom structures such as platinum.^[^ ^22^ ^]^ However, the combination of TiO_2_ structures with HEAs for PEC remains broadly unexplored.

In this context, this study introduces a novel PEC system that combines anodized TiO_2_ nanofilms as the semiconductor substrate with a PtFeCoNiCu HEA as a monolithic catalyst. Using HER performance as the evaluation metric, we elucidate strong synergistic interactions between anodized TiO_2_ nanofilms and HEAs compared to pure titanium thin films, anodized TiO_2_ nanofilms, and HEA films. We further investigate the relationship between photon responsivity and catalytic performance of this hybrid system when the morphology and size of HEAs are finely modified. We also explore how the reaction temperature influences the performance of the proposed PEC system. These results lay a solid foundation for further developments in HEA photoelectrocatalysts, addressing key fundamental questions for the design of highly efficient PEC systems – with implications across a range of reactions and applications.

## 2 Results and Discussion

### 2.1 Superiority of High Entropy Alloys on Titanium Dioxide for PEC‐Driven HER

**Figure 1:**
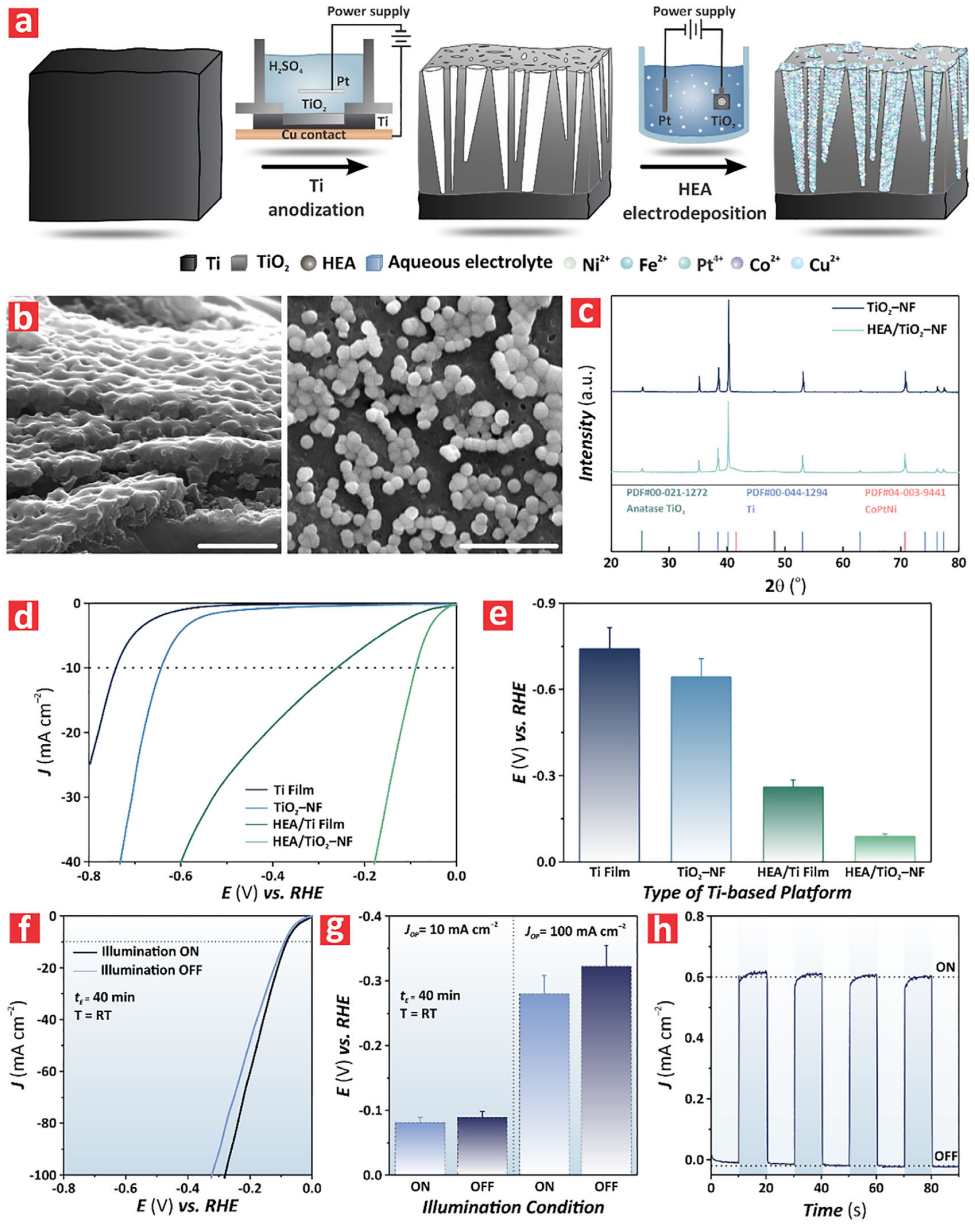
Structural and electrochemical characterization of anodic TiO_2_ nanofilms (TiO_2_–NF) and PtFeCoNiCu HEA/TiO_2_–NF produced by anodization at 120 V and electrodeposition at 1 V for 40 min. a) Schematic image of the fabrication process of PtFeCoNiCu HEA/ TiO_2_–NF via anodization of titanium film at 120 V by 0.5 m sulfuric acid for 3 min to get anodic TiO_2_–NF and electrodeposition process of TiO_2_–NF at 1 V through two‐electrodes system by mixed aqueous solution of metal precursors for 40 min. b) Cross‐sectional (left) and top (right) view FEG‐SEM images of a representative anodic TiO_2_–NF and PtFeCoNiCu HEA/TiO_2_–NF respectively, showing the characteristic morphology of these catalysts (scale bar = 1 and 2 µm, respectively) (NB: data are presented as mean ± SD of a sample size of n ≥ 3 independent measurements). c) XRD spectra of TiO_2_–NF and PtFeCoNiCu HEA/TiO_2_–NF with aligned legends indicating the characteristic peaks of anatase TiO_2_, titanium, and CoPtNi. d) Linear sweep voltammograms of titanium films without any modification, TiO_2_–NF, PtFeCoNiCu HEA/titanium films, and PtFeCoNiCu HEA/TiO_2_–NF under varying overpotential *E*) from –0.8 to 0.0 V versus RHE at a rate of 0.005 V s^−1^ in 1 M KOH. e) Overpotential values in columns versus RHE at *η*_10_ of (d). f) Linear sweep voltammograms of PtFeCoNiCu HEA/TiO_2_–NF fabricated at electrodeposition time for 40 min under illuminated and non‐illuminated conditions and room temperature with varied *E* from –0.8 to 0.0 V versus RHE at a rate of 0.005 V s^−1^ in 1 M KOH. g) Overpotential values in columns versus RHE at *η*_10_ of (f). h) Chronoamperometry of PtFeCoNiCu HEA/TiO_2_–NF produced by anodization at 120 V and electrodeposition at 1 V for 40 min, under ON and OFF illumination modes in 1 M KOH under *E* = 0.0 V versus RHE for a period of 10 s.

**Figure** **1**a illustrates the fabrication process used to deposit PtFeCoNiCu HEA structures on TiO_2_ nanofilms (TiO_2_–NFs). Titanium (Ti) substrates were cut into 1.5 × 1.5 cm chips and anodized in a custom‐built electrochemical reactor with a platinum mesh used as the counter electrode. Anodization of Ti substrates was performed in a 0.5 m sulfuric acid under the application of an anodic voltage of 120 V for 3 min. This process led to the top–down formation of TiO_2_–NFs featuring characteristic pits on the surface, the average size of which was estimated to be 126 ± 13 nm. Subsequently, PtFeCoNiCu HEA was electrodeposited onto the TiO_2_–NFs, using a two‐electrode system under 1 V, where TiO_2_–NFs were the working electrode and a Pt wire mesh was the counter electrode. The deposition electrolyte contained 2 mm of Pt, Fe, Co, Ni, and Cu ion precursors. During the electrodeposition process, the HEA structures grew from the pits to the surface of the TiO_2_–NF, following a Volmer–Weber island growth mechanism, where nanoparticles nucleate, grow, and merge on the surface of TiO_2_–NF with the electrodeposition time.^[^ ^23^, ^24^ ^]^ The empty pits were attributed to the HEA on the TiO_2_–NF surface competing with nearby pits for free metal ions in the solution, leading to the aggregation of larger HEA structures associated with the localization of the electric field across the surface of the semiconductor film. Figure 1a depicts the morphology evolution of HEA growth, from bare TiO_2_–NF to HEA/TiO_2_–NF. Figure 1b (left) presents a cross‐sectional field‐emission scanning electron microscopy (FEG‐SEM) image of a representative TiO_2_–NF fabricated by anodization, revealing the structure of the semiconductor nanofilm featuring pits distributed randomly across its surface. Further FEG‐SEM images are shown in Figure S1 (Supporting Information). Figure 1b (right) shows the top view of the resultant HEA/TiO_2_–NF after 40 min of electrodeposition, where HEA particles grew out of the pit holes and aggregated to form larger clusters on the TiO_2_–NF surface. Figure 1c shows the X‐ray diffraction (XRD) spectra of representative TiO_2_–NFs and HEA/TiO_2_–NFs. This analysis revealed that the as‐produced TiO_2_–NF consisted primarily of titanium dioxide, with minimal diffraction peaks characteristic of the anatase crystal phase observed at 2θ = 25.37° and 48.05°. The formation of small anatase crystals in the as‐produced TiO_2_–NF was attributed to localized Joule heating during high‐voltage anodization.^[^ ^19^ ^]^ The XRD pattern of the HEA/TiO_2_–NF retained similar features to those of the as‐produced TiO_2_–NF because of the incomplete coverage by the surface by HEA particles. However, the diffraction peaks matched the characteristic single‐phase face‐centered cubic (FCC) structure of CoPtNi phases (PDF # 04‐003‐9441).^[^ ^25^ ^]^ Additionally, the weak and broad diffraction peaks observed in the XRD spectra of HEA/TiO_2_–NF were attributed to atomic stacking dislocation within the atomic planes induced by lattice distortion in the PtFeCoNiCu HEA metallene.^[^ ^26^ ^]^ Figure S2 (Supporting Information) shows the Raman spectrum of a model as‐produced TiO_2_–NF and a HEA/TiO_2_–NF after 40 min of electrodeposition, which further demonstrates the presence of anatase TiO_2_.^[^ ^27^ ^]^ In a preliminary set of experiments, we investigated the HER enhancement in the monolithic PtFeCoNiCu HEA/TiO_2_–NF catalysts by performing a comparison with that of a bare titanium (Ti) film, an as‐produced TiO_2_–NF, and a HEA/Ti film (Figure 1d). The analysis revealed that the PtFeCoNiCu HEA/TiO_2_–NF showed the best performance. The current density (*J*) output of the nanofilms was measured under an applied external bias voltage ranging from –0.80 to 0.00 V versus RHE. The overpotential (*E*) required to achieve a current density of *J* = 10 mA cm^−2^ (i.e., *η*_10_) was used as a performance figure of merit for HER. Figure 1e presents a summary of the overpotential values of a Ti film, a TiO_2_–NF, a HEA/Ti film, and a PtFeCoNiCu HEA/TiO_2_–NF at *η*_10_, which were measured to be –0.740 ± 0.074 V, –0.640 ± 0.060, –0.260 ± 0.026, and –0.089 ± 0.0089 V versus RHE, respectively. The estimated difference in overpotential between the HEA/TiO_2_–NF and the other control systems was determined to be 0.65, 0.55, and 0.17 V versus RHE for the Ti film, TiO_2_–NF, and HEA/Ti film, respectively. This result denoted the existence of a cocktail‐like synergistic effect between both components of the hybrid monolithic catalyst (i.e., TiO_2_–NF and HEA nanoparticles), which boosted the HER performance by one order of magnitude. This enhancement could not solely be attributed to the simple addition of independent contributions from the two components. Figure 1f shows the effect of illumination on the HER performance of a HEA/TiO_2_–NF at room temperature in 1 M KOH performed by linear sweep voltammetry (LSV). The HER current density of HEA/TiO_2_–NF under illumination (i.e., ON) demonstrated an enhanced performance associated to photon adsorption by the TiO_2_ film and the additional generation of electron–hole pairs that synergistically contributed to HER efficiency, with extra charge carriers on top of those generated by the externally applied bias on HEA/TiO_2_–NF. Figure 1g provides a comparison of overpotentials at ON and OFF illumination states for the HEA/TiO_2_–NF at *η*_10_ and *η*_100_ of HER. At *η*_10_, the overpotentials of HEA/TiO_2_–NF at ON and OFF states (i.e., ON: –0.081 ± 0.008 V; OFF: –0.089 ± 0.009 V vs RHE) showed a reduced improvement of ≈0.008 V. In contrast, at *η*_100_ the gap of overpotentials of the HEA/TiO_2_–NF increased up to ≈0.04 V (i.e., ON: –0.28 ± 0.03 V; OFF: –0.32 ± 0.03 V vs RHE). It is worth nothing that PtFeCoNiCu HEA is not a semiconductor that can utilize photons to generate electron–hole pairs. Therefore, the increase of overpotential between ON and OFF states, from 0.008 V at *η*_10_ to 0.04 V at *η*_100_, further confirmed the pivotal role of the TiO_2_–NF in the monolithic catalysts to boost HER performance, particularly at high current density range under ON illumination status. This enhancement could be attributed to an improved reduction of photogenerated charge carrier losses in the TiO_2_–NF by the HEA nanoparticles at higher current density, where the effect of the external bias is more effective to extract electrons and holes.^[^ ^28^ ^]^ Figure 1h illustrates the photocurrent generated by the HEA/TiO_2_–NF produced by 40 min of electrodeposition upon sequential ON/OFF illumination cycles at 0 V versus RHE.

This graph clearly demonstrated that these films exhibited an immediate response to the input light stimulus, generating a photoanodic current density significantly higher than the negligible current observed under dark conditions (i.e., *J* ≈ 0 mA cm^−2^ at OFF illumination). The photocurrent difference between light ON and OFF states (i.e., *ΔJ* = *J*_ON_–*J*_OFF_) was measured to be 0.60 ± 0.06 mA cm^−2^.

### 2.2 Chemical and Structural Characterization of HEA/TiO_2_–NFs

**Figure 2:**
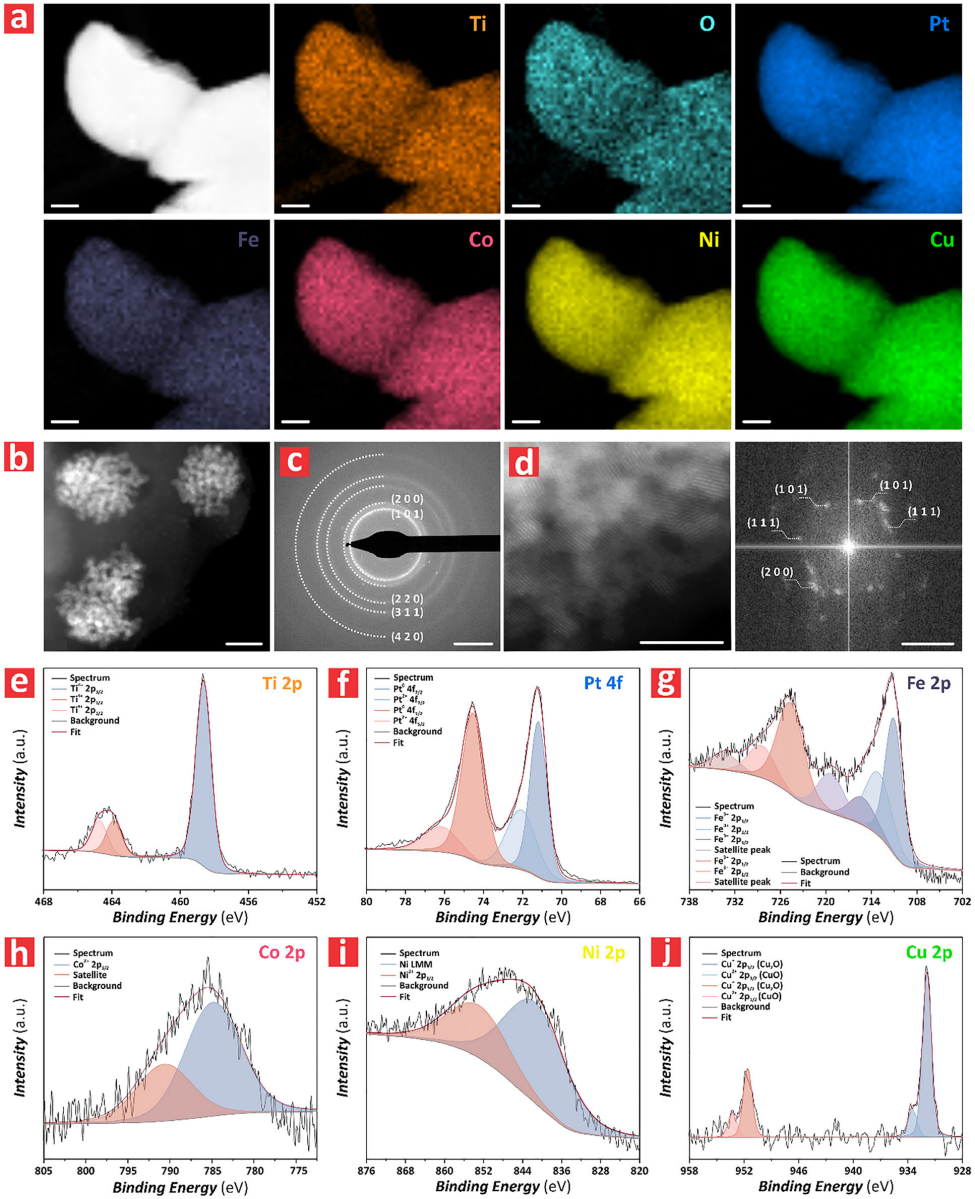
Chemical and structural characterization of PtFeCoNiCu HEA/TiO_2_–NF synthesized via anodization at 120 V and electrodeposition at 1 V for 40 min. a) HAADF‐TEM images of the PtFeCoNiCu HEA/TiO_2_–NF and corresponding EDX element mappings showing the distribution of elements in the PtFeCoNiCu HEA and TiO_2_–NF (scale bars = 5 nm). b) HAADF‐TEM images of the PtFeCoNiCu HEA/TiO_2_–NF, highlighting structural details and features (scale bars = 10 nm). c) SAED patterns of the PtFeCoNiCu HEA/TiO_2_–NF, confirming crystallographic features (scale bars = 5 nm^−1^). d) HAADF‐TEM images (left) of the PtFeCoNiCu HEA/TiO_2_–NF and the corresponding FFT patterns (right) to further verify the structural phases of the monolithic PEC system (scale bars = 5 nm and 5 nm^−1^, respectively). e–j) High resolution XPS spectra of: (e) Ti 2p, (f) Pt 4f, (g) Fe 2p, h) Co 2p, i) Ni 2p, and j) Cu 2p of the PtFeCoNiCu HEA/TiO_2_–NF system.

To identify HER enhancements associated with the structure and composition of the HEA/TiO_2_–NF system, we performed a comprehensive set of characterizations to gain insights into the key features of this PEC platform. **Figure** **2**a presents the high‐angle annular dark‐field scanning transmission electron microscopy (HAADF‐STEM) images along with corresponding EDX element mapping images of a PtFeCoNiCu HEA/TiO_2_–NF synthesized by electrodeposition of PtFeCoNiCu from aqueous precursors onto the TiO_2_–NF. The EDX element mapping images revealed that Pt, Fe, Co, Ni, and Cu were uniformly distributed within the structure of the HEA particles, whereas Ti and O were found to be uniformly distributed in the TiO_2_–NF substrate. Figure S3 (Supporting Information) provides further confirmation of the morphology and homogeneous distribution of Pt, Fe, Co, Ni, and Cu in the HEA/TiO_2_–NF system by EDX mapping performed during FEG‐SEM characterization. Figure 2b shows an HAADF‐STEM image of the HEA/TiO_2_–NF over an extended area, where the brighter regions corresponded to the HEA particles, and the relatively darker regions featuring porous structures were the TiO_2_–NF substrate. Figure 2c illustrates the selected area electron diffraction (SAED) patterns of the PtFeCoNiCu HEA/TiO_2_–NF with the characteristic XRD patterns, which further confirmed the single‐phase FCC structure of HEA and the anatase phase of TiO_2_ in the TiO_2_–NF substrate. Figure 2d (left) provides an HAADF‐STEM image of the edge of a HEA/TiO_2_–NF. Because of the extended thickness of the TiO_2_–NF (i.e., 225 ± 23 nm of anodic film over the titanium substrate), the feasibility of fast Fourier transform (FFT) analysis over the extended interface of both systems was limited. To overcome this, a more marginal area was selected for imaging. Figure 2d (right) shows the FFT patterns of the magnified left image, which clearly confirms the FCC structure of the HEA and the anatase phase of the TiO_2_–NF. Additionally, Figure S4 (Supporting Information) further supported these findings, corroborating the correlation between XRD and SAED results. X‐ray photoelectron spectroscopy (XPS) was performed to characterize the electronic states and metallic elements of the HEA/TiO_2_–NF. Figure 2e–j presents the XPS spectra of Ti 2p, Pt 4f, Fe 2p, Co 2p, Ni 2p, and Cu 2p, respectively, and Figure S5 (Supporting Information) shows the full XPS spectrum of the HEA/TiO_2_–NF. These analyses confirmed the presence of elements that were consistent with the composition of the HEA/TiO_2_–NF obtained by EDX element mapping images (vide supra). Figure 2e displays the XPS spectrum of Ti 2p, where the peak at ≈464.60 eV corresponds to the TiO_2_ substrate, whereas the peak at 458.60 eV was attributed to the anatase phase of TiO_2_ associated with localized Joule heating during high‐voltage anodization.^[^ ^29^, ^30^ ^]^ Figure 2f shows the XPS spectrum of Pt 4f, with the peak at 71.30 eV indicating metallic Pt, and the peak at 74.65 eV being attributed to the Ti atom from TiO_2_, potentially indicating the formation of Pt_3_Ti.^[^ ^31^, ^32^ ^]^ Figure 2g illustrates the XPS spectrum of Fe 2p, with the peak at 711.10 eV corresponding to Fe/Ni and the peak at 724.20 eV to Fe/Cu, highlighting the coordination environment of Fe in the HEA.^[^ ^33^, ^34^ ^]^ The peaks of Co and Ni in Figure 2h,i were at ≈785.5 and 847.0 eV, respectively, which differed from their corresponding metallic peaks expected at 778.2 and 852.6 eV. This shift in peak positions could be associated with the altered local chemical environment of Co and Ni atoms due to the mixing of different elements in the HEA. This distortion could lead to a redistribution of electron density and cause shifts in the binding energy of core levels, thereby explaining the observed XPS peak shifts. The XPS peaks of Co and Ni were also consistent with the XRD patterns of the CoPtNi alloy. Figure 2j represents the XPS spectra of Cu 2p, of which the peak at 952.93 eV corresponded to metallic Cu, whereas the peak at 932.01 eV was associated with Cu/Ni, suggesting that Cu atoms were influenced by neighboring Ni atoms in the HEA.^[^ ^35^, ^36^ ^]^

### 2.3 Enhancement of HER Performance by Electrodeposition Time in HEA/TiO_2_–NFs

**Figure 3:**
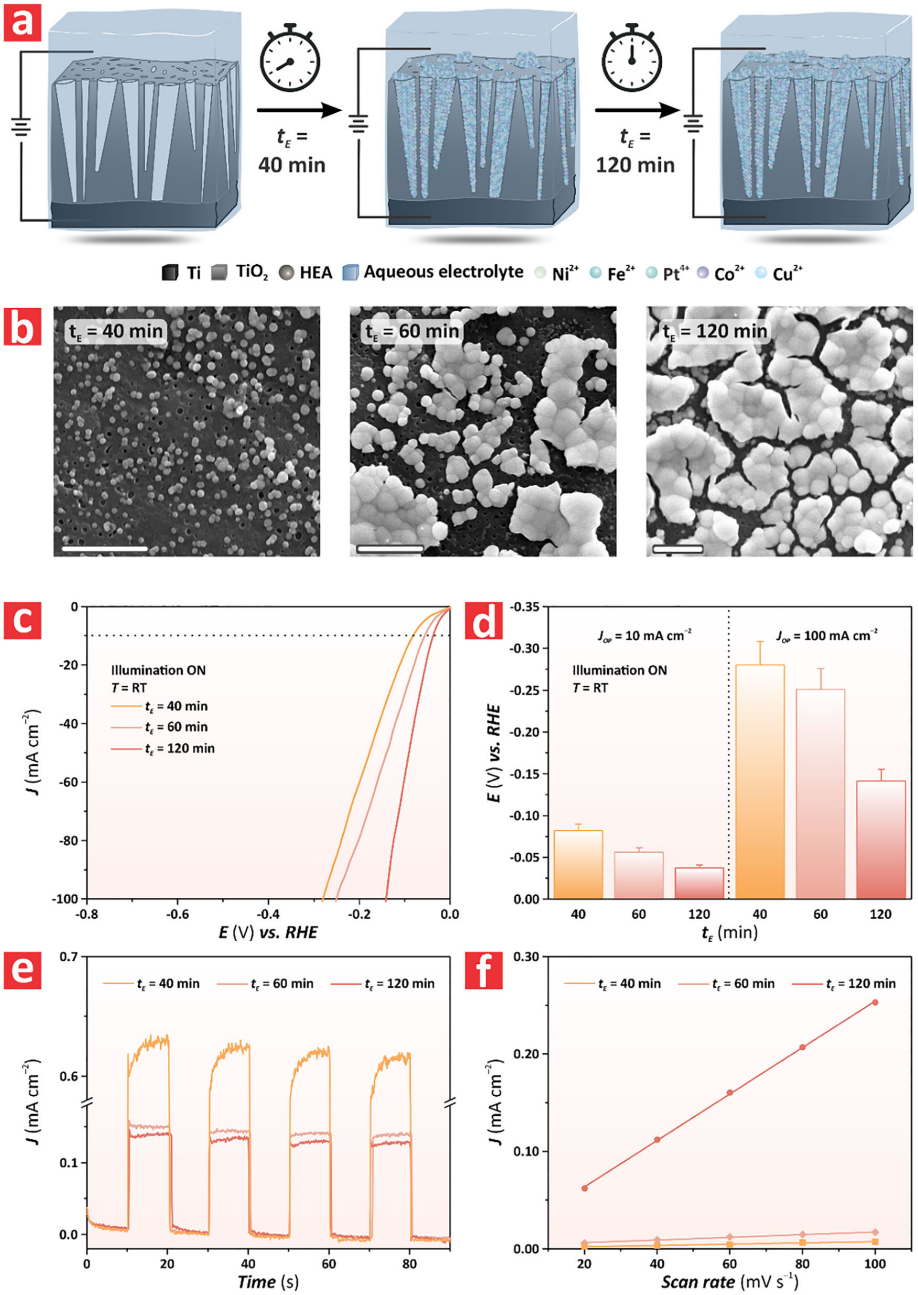
Structural and electrochemical characterization of PtFeCoNiCu HEA/TiO_2_–NF synthesized via anodization at 120 V and electrodeposition at 1 V for 40, 60, and 120 min. a) Schematic of the electrodeposition process used to fabricate PtFeCoNiCu HEA/TiO_2_–NFs at 1 V in a two‐electrode system from a mixed aqueous solution of metal precursors for 40, 60, and 120 min. b) Top‐view FEG‐SEM images of a PtFeCoNiCu HEA/TiO_2_–NF fabricated at distinct electrodeposition times (i.e., from left to right: *t*_E_ = 40, 60, and 120 min, respectively), showing the characteristic morphology of these PEC films (scale bars = 2.5 µm). c) Linear sweep voltammograms of PtFeCoNiCu HEA/TiO_2_–NFs synthesized at electrodeposition times of 40, 60 and 120 min under varying *E*, from –0.8 to 0.0 V versus RHE, at a rate of 0.005 V s^−1^ in 1 M KOH. d) Summary of overpotential values versus RHE at *η*_10_ measured in (c) (NB: data are presented as mean ± SD of a sample size of n ≥ 3 independent measurements). e) Chronoamperometry of PtFeCoNiCu HEA/TiO_2_–NFs produced by anodization at 120 V and electrodeposition at 1 V for 40, 60, and 120 min under ON and OFF illumination modes in 1 M KOH under an *E* bias of 0.0 V versus RHE for a period of 10 s. f) ECSA of PtFeCoNiCu HEA/TiO_2_–NFs produced by anodization at 120 V and electrodeposition at 1 V for 60 and 120 mins (NB: data are presented as mean ± SD of a sample size of n ≥ 3 independent measurements).

Our observations indicated that the electrodeposition duration significantly determined the morphology and size of the HEA particles synthesized on the surface of the TiO_2_–NFs. Motivated by this, we decided to analyze the effect of these features on the HER performance of the PtFeCoNiCu HEA/TiO_2_–NF system by systematically varying the electrodeposition time, from 40 to 120 min. **Figure** **3**a provides schematic representations of the idealized PtFeCoNiCu HEA/TiO_2_–NF system at distinct electrodeposition times (i.e., 40, 60, and 120 min). During the electrodeposition process, HEA‐based structures started filling the pits on the surface of the TiO_2_–NF by forming nanoparticles. Upon further extension of the deposition time, the HEA particles filled completely the pits and started forming larger particles in the form of islands on the surface of the TiO_2_–NF. As the process continued, the particles grew further and extended over the surface of the film following a mechanism that could be approximated to a Volmer–Weber island growth. Figure 3b shows top‐view FEG‐SEM images of the HEA/TiO_2_–NFs synthesized at electrodeposition times (*t*_E_) of 40, 60, and 120 min. At *t*_E_ = 40 min, the HEA structures on the surface of the HEA/TiO_2_–NFs were in the form of uniformly distributed nanoparticles with an average size of 0.17 ± 0.02 µm. The surface of the TiO_2_–NFs remained visible underneath. As the deposition time increased, the HEA nanoparticles started to aggregate and form larger HEA particles or islands, the average size of which was determined to be 3.2 ± 0.32 µm. After 120 min, the HEA particles were substantially larger with an average size of 3.5 ± 0.35 µm and almost covered the entire surface of the TiO_2_–NF. Figure S6 (Supporting Information) presents the Raman spectra of the PtFeCoNiCu HEA/TiO_2_–NF at *t*_E_ = 40 and 120 min. These spectra show the characteristic Raman bands of TiO_2_–NFs at 145 and 639 cm^−2^. The reduced peak intensity of the HEA/TiO_2_–NF at *t*_E_ = 120 min compared to that of the same film at *t*_E_ = 40 min would suggest that the larger HEA particles blocked the Raman signal from the underlying TiO_2_–NF substrate. Figure 3c provides a comparison of the HER performance of the HEA/TiO_2_–NF system at *t*_E_ = 40, 60 and 120 min under illumination (ON state), indicating an enhancement in HER efficiency (i.e., reduction in overpotential) with *t*_E_, from 40 to 120 min, where the HEA/TiO_2_–NF system fabricated at *t*_E_ = 120 min achieved the best performance. Figure 3d summarizes the overpotentials at ON illumination states for these HEA/TiO_2_–NFs at *η*_10_ and *η*_100_ of HER. At *η*_10_, the overpotentials of HEA/TiO_2_–NFs produced at *t*_E_ = 40, 60, and 120 min were –0.081 ± 0.008, –0.056 ± 0.006, and –0.037 ± 0.004 V versus RHE, respectively. At *η*_100_, the overpotentials of these HEA/TiO_2_–NFs increased slightly, with measured values of –0.280 ± 0.030, –0.250 ± 0.030, and –0.014 ± 0.01 V versus RHE, respectively. The HER performance under non‐illuminated conditions (i.e., OFF state) of the HEA/TiO_2_–NFs fabricated at *t*_E_ = 40, 60, and 120 min is shown in Figure S7 (Supporting Information). It was apparent that these followed the same trend as that shown in the ON state of illumination, but with reduced efficiency (vide infra). This further corroborated the key contribution of the semiconductor TiO_2_–NF with the generation of additional charge carriers to drive HER. Figure S8 (Supporting Information) compares the HER performance at *η*_10_ and *η*_100_ of a model HEA/Ti film produced at *t*_E_ = 120 min and that of a HEA/TiO_2_–NF fabricated at the same electrodeposition time. The recorded overpotentials under these conditions were –0.076 ± 0.008 and –0.056 ± 0.006 V versus RHE at *η*_10_, and –0.19 ± 0.02 and –0.14 ± 0.01 V versus RHE at *η*_100_, respectively. It is apparent that HEA/TiO_2_–NF attained superior HER performance because of the synergistic effect between the HEA and the semiconductor film, which further corroborated our initial findings. Figures S9 and S10 (Supporting Information) examine the overpotential difference between illuminated and non‐illuminated conditions (*ΔE*_ON–OFF_) at *η*_10_ and *η*_100_ of HER for the HEA/TiO_2_–NFs produced at *t*_E_ = 40, 60, and 120 min. The average value of *ΔE*_ON–OFF_ at *η*_10_ was measured to be ≈10 mV for all the analyzed HEA/TiO_2_–NFs. However, at *η*_100_ *ΔE*_ON–OFF_ decreased substantially from ≈4.9 mV for the HEA/TiO_2_–NF produced at *t*_E_ = 60 min to 1.7 mV recorded for its counterpart fabricated at *t*_E_ = 120 min. We further investigated this phenomenon by analyzing the photocurrent upon sequential ON/OFF illumination cycles at 0 V versus RHE generated by the HEA/TiO_2_–NFs produced at *t*_E_ = 40, 60, and 120 min (Figure 3e). It was demonstrated that the HEA/TiO_2_–NF produced at *t*_E_ = 40 min exhibited higher light responsiveness than that of its analogue films fabricated at *t*_E_ = 60 and 120 mins. Figure S11 (Supporting Information) summarizes the overpotentials at 0 V versus RHE of PtFeCoNiCu HEA/TiO_2_–NFs synthesized at electrodeposition times of 40, 60, and 120 min under ON and OFF illumination states at 10 s intervals, showing a decreasing trend in light responsiveness, from 0.62 to 0.12 mA cm^−2^ from *t*_E_ = 40 to 120 min, respectively. However, despite the reduced photoactivity, our previous results revealed that the HEA/TiO_2_–NF produced at *t*_E_ = 120 min outperformed its counterpart HEA/TiO_2_–NF fabricated at *t*_E_ = 60 mins in HER. To investigate the reason for this difference, Figure 3f illustrates the electrochemical surface area (ECSA) of both PEC films in terms of the slope of double‐layer capacitance. This analysis indicated that the HEA/TiO_2_–NF fabricated at *t*_E_ = 120 min had larger ECSA than that of its counterparts HEA/TiO_2_–NFs produced at *t*_E_ = 40 and 60 min, with quantified values of 0.065, 0.14, and 2.4 µF cm^−2^, respectively. Figure S12 (Supporting Information) shows the cyclic voltammetry of these films and the corresponding ECSA values obtained by dividing the double‐layer capacitance by the specific capacitance of these films, the value of which was determined to be 75.57, 159.01, and 2779.07 cm^2^ cm^−2^, respectively. These findings demonstrated that extending the electrodeposition time enhances HER performance by increasing the HEA particle size and providing more active sites and a larger active surface area for HER, despite the slight reduction in light responsiveness. As such, this would indicate that the electrocatalytic component was the dominant factor contributing to the PEC‐driven HER in the HEA/TiO_2_–NF system. Another important aspect to consider is the individual contribution of the photocatalyst (i.e., TiO_2_) and electrocatalyst (i.e., HEA) components of the HEA/TiO_2_–NF photoelectrocatalyst on the overall PEC performance. To this end, we analyzed the photocurrent density of a model HEA/TiO_2_–NF under three conditions: (i) no external potential bias under ON illumination state (PC); (ii) external potential bias under OFF illumination state; and (iii) external potential bias under ON illumination state. The photocurrent density quantified in the HEA/TiO_2_–NF was measured to be –1.140 ± 0.10, 0.140 ± 0.10, and 0.02 ± 0.01 mA cm^−2^, respectively. A summary of the results is presented in Figure S13 (Supporting Information). We further analyzed the effect of light intensity on the overall performance of the photoelectrocatalyst. Analysis of the photocurrent obtained under three different light illumination intensities (i.e., 120, 150, and 200 W) revealed that the higher the illumination intensity the higher the photocurrent density was, with average values of –1.10 ± 0.10, –1.13 ± 0.10, and 1.21 ± 0.10 mA cm^−2^ at 120, 150, and 200 W, respectively (Figure S14, Supporting Information). In terms of overpotential, it was found that a change in light intensity from 200 to 150 W resulted in a slight increase of overpotential, from –0.13 ± 0.10 to –0.14 ± 0.10 V versus RHE (Figure S15, Supporting Information).

### 2.4 Enhancement of HER Performance in HEA/TiO_2_–NFs by Thermal Stimulation

**Figure 4:**
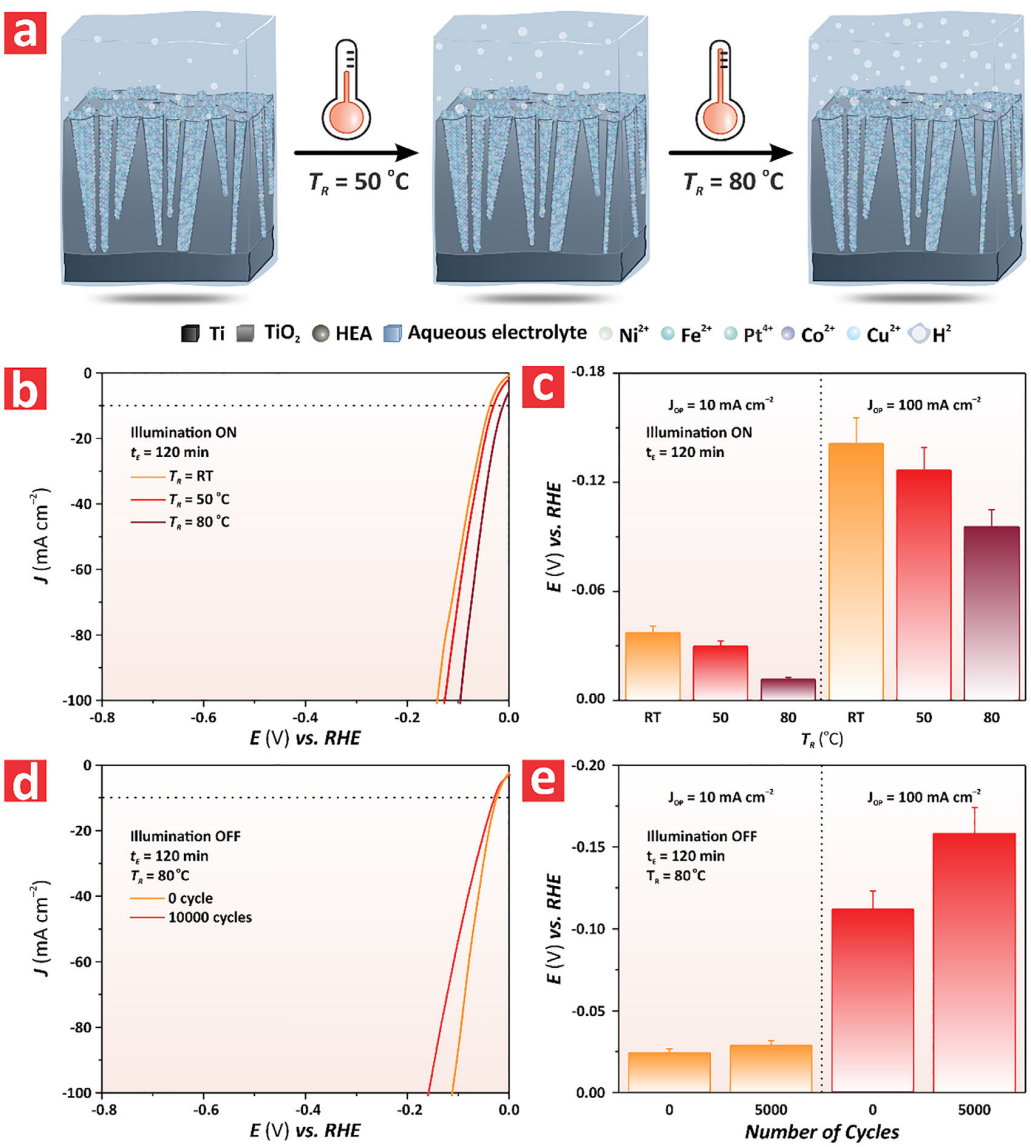
Electrochemical characterization of PtFeCoNiCu HEA/TiO_2_–NF synthesized via anodization at 120 V and electrodeposition at 1 V for 120 min at different reactant electrolyte temperature, from room temperature (RT) to 80 °C. a) Schematic of the PtFeCoNiCu HEA/TiO_2_–NF fabricated by via anodization at 120 V and electrodeposition at 1 V for 120 min at varied reactant electrolyte temperature. b) Linear sweep voltammograms of the PtFeCoNiCu HEA/TiO_2_–NF synthesized by anodization at 120 V and electrodeposition at 1 V for 120 min at *T*_R_ = RT, 50 and 80 °C under ON illumination state and varying *E*, from –0.8 to 0.0 V versus RHE at a rate of 0.005 V s^−1^ in 1 M KOH. c) Summary of overpotential values in terms of *E* versus RHE at *η*_10_ and *η*_100_ extracted from (b). d) Linear sweep voltammograms of the PtFeCoNiCu HEA/TiO_2_–NF synthesized by anodization at 120 V and electrodeposition at 1 V for 120 min at 0 and 10 000 HER cycles in 1 M KOH under OFF illumination state. e) Summary of overpotential values versus RHE at *η*_10_ and *η*_100_, measured in d) (NB: data are presented as mean ± SD of a sample size of n ≥ 3 independent measurements).

The combination of anodization and electrodeposition – both fully scalable, cost‐competitive fabrication technologies – presents potential opportunities to develop state‐of‐the‐art photoelectrocatalysts for real‐life applications. Therefore, to further enhance HER efficiency and better align PtFeCoNiCu HEA/TiO_2_–NFs with practical production requirements, we analyzed the impact of elevated electrochemical reaction temperature on the HER performance. **Figure** **4**a shows the schematics of PtFeCoNiCu HEA/TiO_2_–NFs exposed to electrolytes at distinct reaction temperatures (i.e., *T*_R_ = room temperature, 50, and 80 °C) to generate more hydrogen gas via PEC. Figure 4b summarizes the HER performance of HEA/TiO_2_–NFs synthesized via anodization at 120 V and electrodeposition at 1 V for 120 min under ON illumination state and increasing *T*_R_, from room temperature (RT) to 80 °C. At the *E* = 0 V versus RHE, the current density generated by the model HEA/TiO_2_–NF increased from ≈0 to –6 mA cm^−2^, indicating an HER enhancement stimulated by the photothermal effect. According to the improved Varshni equation, the bandgap of a semiconductor is dependent on the temperature.^[^ ^37^ ^]^ It is also known that the bandgap of TiO_2_ decreases at high temperatures.^[^ ^38^ ^]^ The increase in the reactant electrolyte temperature may cause oxygen loss in the TiO_2_ and an increase in oxygen vacancies that can boost HER performance.^[^ ^39^ ^]^ Another potential contribution could be localized surface plasmon resonances (LSPR) generated in the HEA metal nanoparticles upon illumination, associated with their metallic nature, which could contribute to the overall enhancement of HER by boosting excitation and transfer of charge carriers, catalytic reaction kinetics, and a decrease in apparent activation energy.^[^ ^40^ ^]^ Figure S16 (Supporting Information) shows the reflectance spectra of PtFeCoNiCu HEA/TiO_2_–NFs synthesized at electrodeposition times of 40, 60, and 120 min, where it can be clearly seen a characteristically broad LSPR band at ≈955 nm. This analysis also revealed that the bandwidth of the band increased with the increasing size of the HEA structures by the electrodeposition time, which is in good agreement with the increasing particle size of the HEA. Figure 4c illustrates the HER performance of the HEA/TiO_2_–NFs in terms of overpotential at *η*_10_ and *η*_100_ of HER obtained from Figure 4b. Analysis of this figure of merit at *T*_R_ = RT, 50, and 80 °C at *η*_10_ revealed that the overpotential decreased progressively with *T*_R_, with quantified values of –0.037 ± 0.004, –0.030 ± 0.003, and –0.011 ± 0.001 V versus RHE, respectively. At *η*_100_, the overpotentials of the HEA/TiO_2_–NF at *T*_R_ = RT, 50 and 80 °C were –0.140 ± 0.010, –0.130 ± 0.010, and –0.096 ± 0.010 V versus RHE, respectively. It was apparent from these results that the increased reactant electrolyte temperature significantly enhanced HER performance. Figure S17 (Supporting Information) shows the LSV of the HEA/TiO_2_–NF synthesized via anodization at 120 V and electrodeposition at 1 V for 120 min under the OFF illumination state, demonstrating an enhancement in HER performance driven by photothermal effect, which was consistent with Figure 4b when the current density of the model HEA/TiO_2_–NF increased from ≈0 to –6 mA cm^−2^ at *E* = 0 V versus RHE. Figures S18 and S19 (Supporting Information) show a comparison of HER performance in terms of overpotentials at *η*_10_ and *η*_100_ under ON and OFF illumination states, indicating that the HER was more active under ON illumination state. This analysis also revealed that *ΔE*_ON–OFF_ at *η*_10_ and *η*_100_ decreased with increasing temperature, from *T*_R_ = RT to 80 °C (Figure S20, Supporting Information). This phenomenon was attributed to the more efficient excitation of electron–hole pairs during the gradual electrolyte temperature increase, facilitated by photothermal effects and LSPR, which in turn reduced the required overpotential to drive HER under ON illumination state. We further demonstrated the potential of the PtFeCoNiCu HEA/TiO_2_–NF PEC system for real‐life applications by analysing the durability and performance reproducibility of this model material over multiple HER cycles. Figure 4d presents the stability test consisting of 10 000 HER cycles under non‐illumination conditions at a reactant electrolyte temperature of 80 °C, indicating slight attenuation of overpotential after 10 000 cycles, demonstrating the potential for long‐term and industry use of HEA/TiO_2_–NFs.

Figure 4e summarizes the HER performance at *η*_10_ and *η*_100_ of the HEA/TiO_2_–NF before and after 10 000 HER cycles under OFF illumination states. At *η*_10_, the overpotentials decreased from –0.024 ± 0.002 to –0.029 ± 0.003 V versus RHE, respectively. At *η*_100_, the overpotentials decreased from –0.11 ± 0.01 to –0.16 ± 0.02 versus RHE, respectively. The slight increase of overpotential after 10 000 cycles of HER was attributable to the prolonged exposure to high‐temperature, strongly alkaline electrolyte conditions, resulting in partial deactivation and agglomeration of the catalyst, leading to the loss of active sites and subsequent performance degradation. The structural integrity of the HEA/TiO_2_–NF before and after 5000 HER cycles was analyzed via FEG‐SEM, and no sign of structural change was observed (Figure S21, Supporting Information). We further analyzed the stability of the HEA/TiO_2_–NF by chronopotentiometric assessment over a period of 100 h at a constant current density input of 100 mA cm^−2^ (Figure S22, Supporting Information). The overpotential quantified over time remained constant, demonstrating that the HEA/TiO_2_–NF system can maintain a constant overpotential over this period of time under harsh HER working conditions.

### 2.5 HER Enhancement Mechanism in HEA/TiO_2_–NFs via In Situ Raman and Simulations

**Figure 5:**
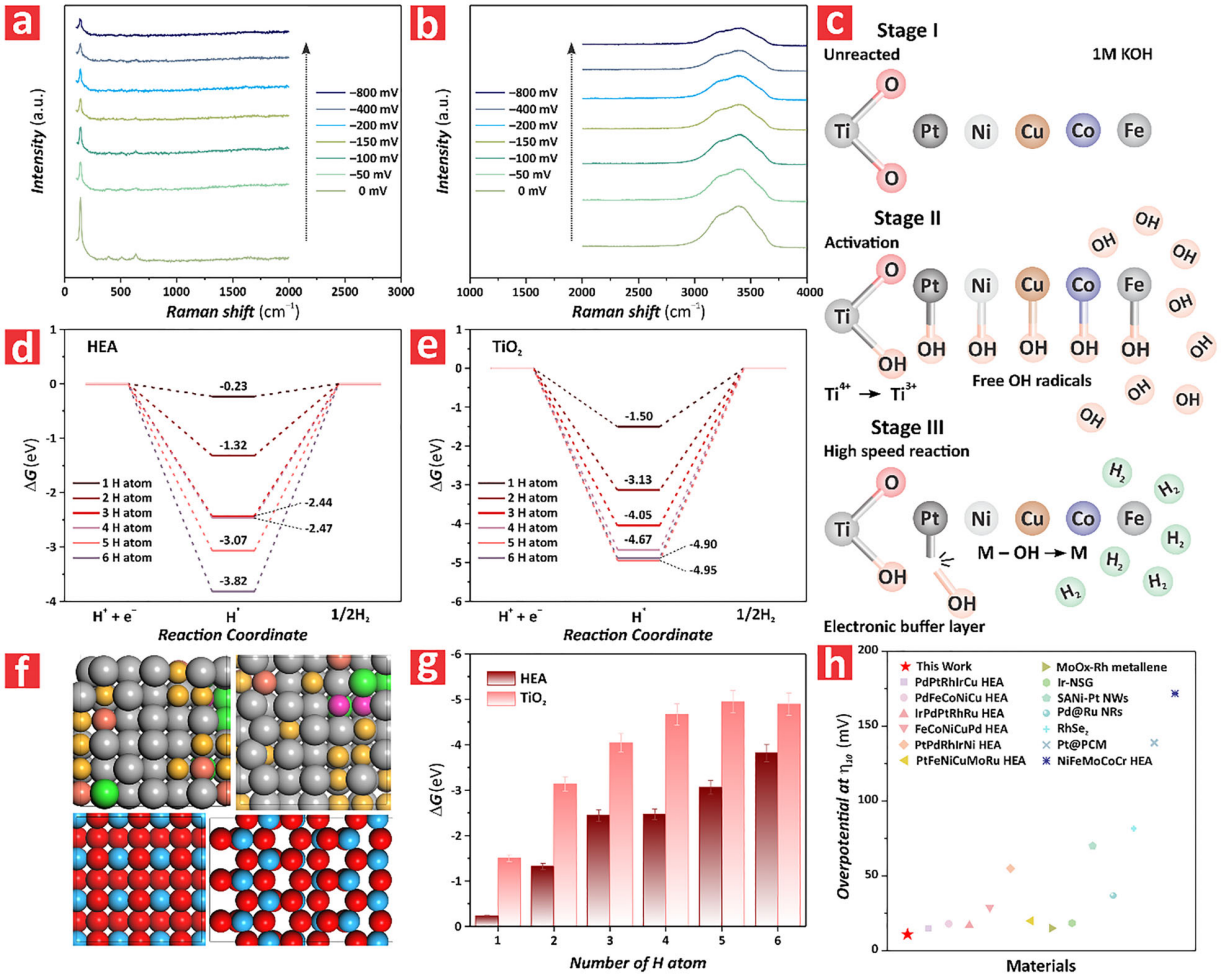
Unravelling the HER mechanism in PtFeCoNiCu HEA/TiO_2_–NFs synthesized via anodization at 120 V and electrodeposition at 1 V for 120 min. a) Electrochemical in situ Raman spectra of a model PtFeCoNiCu HEA/TiO_2_–NF during HER in 1 M KOH electrolyte at room temperatures under increasing potential bias, from 0 to –800 mV versus RHE and from 100 to 2000 cm^−1^. b) Electrochemical in situ Raman spectra of a model PtFeCoNiCu HEA/TiO_2_–NF during HER in 1 M KOH electrolyte at room temperatures under increasing potential bias, from 0 to –800 mV versus RHE and from 2000 to 4000 cm^−1^. c) Idealized structure of a PtFeCoNiCu HEA/TiO_2_–NF during the different HER reaction stages under increasing overpotential in 1 M KOH electrolyte. d) Gibbs free energy of the HER pathway at increasing H atoms, from 1 to 6, on PtFeCoNiCu HEA. e) Gibbs free energy of H adsorption and desorption (*ΔG*) at increasing H atoms, from 1 to 6, on anatase TiO_2_. f) Top and side view schematics of the atomic structure of PtFeCoNiCu HEA (top) and anatase TiO_2_ (bottom) used in our DFT simulations. g) Comparison of required Gibbs free energy of H adsorption between PtFeCoNiCu HEA and anatase TiO_2_, from 1 to 6 adsorbed H atoms. h) State‐of‐the‐art comparison of HER performance at *η*_10_ between our PtFeCoNiCu HEA/TiO_2_–NF system and other comparable studies.

To unravel the HER reaction mechanism in PtFeCoNiCu HEA/TiO_2_–NFs and establish a mechanistic framework that explained our experimental observations, we performed in situ Raman analysis and density function theory (DFT) simulations. **Figure** **5**a shows the in situ Raman spectra, from 100 to 2000 cm^−1^, of a model PtFeCoNiCu HEA/TiO_2_–NF in 1 M KOH under applied potentials from open circuit potential (i.e., *E* = 0 mV vs RHE) to –800 mV versus RHE. The reaction consisted of three stages: (i) unreacted stage; (ii) activation stage; and (iii) high‐speed stage. The first stage was performed at open circuit potential, and four characteristic Raman bands corresponding to the anatase phase of TiO_2_ (i.e., ≈144, 393, 512, and 633 cm^−1^) were clearly observed, indicating a well‐ordered crystal lattice structure. As the potential was negatively increased up to –150 mV versus RHE during the HER activation stage, the intensities of the Raman bands gradually diminished. This attenuation of the Raman signal was associated with the progressive distortion of the crystal lattice of TiO_2_, where Ti⁴⁺ underwent a reduction to its Ti^3^⁺ oxidation state.^[^ ^41^, ^42^ ^]^ This resulted in an enhancement of the interfacial charge transfer, where TiO_2_ became an electronic buffer layer. When the external potential was further increased up to –800 mV versus RHE during the high‐speed stage, the intensity of the Raman bands decreased dramatically, and only the 144 cm^−1^ band was observable. This would suggest a possible hydrogen intercalation within the TiO_2_ lattice, contributing to the loss of Raman selection rules.^[^ ^43^, ^44^ ^]^ The gradual attenuation and eventual vanishment of the TiO_2_ Raman bands under increasing cathodic potentials indicated that the TiO_2_ underwent significant lattice distortion, Ti^3^⁺ formation, and interfacial charge transfer. This dynamic evolution highlighted the synergistic mechanism between HEA and TiO_2_, wherein the HEA dominated the H* adsorption and hydrogen evolution, while TiO_2_ acted as an electron buffer, which underwent a dynamic restructure during the reaction. Figure 5b shows the in situ Raman spectra, from 2000 to 4000 cm^−1^, of PtFeCoNiCu HEA/TiO_2_–NF in 1 M KOH electrolyte under applied potentials from open circuit potential (i.e., *E* = 0 mV vs RHE) to –800 mV versus RHE. The Raman bands located at 3000 to 3700 cm^−1^ corresponded to various O–H stretching modes arising from the adsorbed water molecules, hydrogen‐bonded networks, and surface hydroxyl species (M–OH, Ti–OH). Magnified spectra showing the Raman bands are shown in Figures S23 and S24 (Supporting Information). Upon increasing cathodic potential, these bands progressively weakened and eventually vanished from the spectra, indicating a continuous depletion or reorganization of interfacial water layers and hydroxyl functionalities. This would suggest that the reduction of metal–OH bonds (e.g., Pt–OH, Ni–OH, Cu–OH, Fe–OH, and Co–OH) to metallic states facilitated by the hydrogen evolution, as indicated by the disappearance of OH‐related Raman features. Notably, the progressive loss of OH– and H_2_O‐related Raman features under increasing cathodic bias was not followed by a concurrent suppression of HER activity. Rather, it reflected a rapid turnover of interfacial species – namely, water adsorption, dissociation, and hydrogen evolution – that led to a lower steady‐state coverage of vibrationally active surface intermediates. This was consistent with the accelerated HER kinetics at higher overpotentials, wherein surface‐bound species were quickly consumed in the catalytic cycle. Therefore, the observed spectral attenuation substantiated the enhanced catalytic kinetics, rather than surface passivation. These results underscored the dynamic and responsive nature of the HEA–TiO_2_ interface under alkaline HER conditions. The loss of vibrationally active surface species was aligned with the accelerated turnover of interfacial reactants, revealing an inherently efficient water dissociation capability and strong electronic interaction between the HEA and the semiconductor support. This in situ spectroscopic evidence provided direct insights into the real‐time evolution of active sites and interfacial structures during the catalytic process. Although further analyses will be needed to fully determine the enhancement mechanism, our results are in good agreement with recent studies using advanced spectroscopic techniques analyzing synergies between HEAs and semiconductors for photocatalysis.^[^ ^45^ ^]^ These reports indicated a combination of Schottky junctions, efficient charge separation, prolonged carrier lifetime, and localization of electron accumulation and density on highly active Pt sites, and the formation of Pt‐based bridges for optimal hydrogen binding.

Figure 5c illustrates a proposed model for HER on PtFeCoNiCu HEA/TiO_2_–NFs in 1 M KOH with increased overpotentials, from 0 to –800 mV, where the three stages of reaction occur on the PEC surface according to the in situ Raman spectra analysis shown in Figure 5a,b. Stage I represents the non‐reactive phase, where TiO_2_ retained its intact lattice structure and the PtFeCoNiCu HEA was located on the TiO_2_–NF without initiating catalytic activity. In Stage II, TiO_2_ was activated and started to function as an electronic buffer layer, facilitating interfacial charge transfer to enhance the hydrogen evolution reaction. Meanwhile, the PtFeCoNiCu HEA acted as the primary HER active sites, initiating adsorption and partial dissociation of water molecules on the catalyst surface. However, the overall HER rate remained relatively low during this stage. In Stage III, at higher overpotentials, the HER process entered a high‐activity regime. Water molecules were rapidly adsorbed and consumed at the catalyst interface, accelerating the turnover of reaction intermediates such as OH radicals. This led to reduced surface coverage of vibrationally active species, increased hydrogen generation rates, and enhanced catalytic efficiency. The synergistic interaction between the HEA and the dynamically restructured TiO_2_ further boosted the reaction kinetics. Figure S25 (Supporting Information) illustrates the full range of in situ Raman spectra, from 0 to 4000 cm^−1^, for the PtFeCoNiCu HEA/TiO_2_–NF. Figure 5d compares the simulated Gibbs free energy of H adsorption (*ΔG*) on PtFeCoNiCu HEA by adsorbing H atoms, from 1 to 6 H atoms, with corresponding atomic structure images. These simulations revealed an increment in *ΔG*, from –0.23 to –3.82, when the adsorbed H atoms increased from 1 to 6. We also calculated the *ΔG* values for anatase TiO_2_ upon the adsorption of an increasing number of H atoms, from 1 to 6, along with the corresponding atomic structure images of this system (Figure 5e). This analysis indicated an increment of ΔG, from –1.5 to –4.95, when the number of H atoms adsorbed increased from 1 to 6. Figure 5f shows the top and side view atomic structures of the PtFeCoNiCu HEA and anatase crystal of TiO_2_ used for DFT simulations. Figure 5g illustrates a comparison the H adsorption and desorption energy of PtFeCoNiCu HEA and anatase TiO_2_, from 1 H atom to 6 H atoms, revealing that the PtFeCoNiCu HEA achieved higher catalytic reactivity than that of anatase TiO_2_ due to the value of *ΔG* closer to 0, which denotes that H is adsorbed and desorbed more easily to form H_2_.^[^ ^46^ ^]^ Figures S26–S31 (Supporting Information) provide a comparison of *ΔG* of PtFeCoNiCu HEA and anatase TiO_2_, from 1 H atom to 6 H atoms, and the corresponding structures with H atom adsorption sites. Tables S1 and S2 (Supporting Information) provide the *ΔG*_ad_ of PtFeCoNiCu HEA and anatase TiO_2_, from 1 H atom to 6 H atoms with increased reaction temperatures, from room temperature to 80 °C, showing that the *ΔG*_ad_ remained at a stable value during higher temperature reaction, confirming the great thermal stability. The simulation data corroborated that the PtFeCoNiCu HEA had higher catalytic activity and H adsorption capacity than that of anatase TiO_2_. Although anatase TiO_2_ featured lower catalytic activity than PtFeCoNiCu HEA, anatase TiO_2_ can provide more active sites if combined with PtFeCoNiCu HEA. Hence, the combination of PtFeCoNiCu HEA and anatase TiO_2_ increased the total active sites, since TiO_2_ can adsorb the higher energy requirements for H atom, compensating the shortcoming of PtFeCoNiCu HEA, which only contains lower energy required for active sites. We further extended this analysis by estimating the Gibbs free energy of the PtFeCoNiCu HEA from water adsorption and ^*^H_2_O dissociation to ^*^OH and ^*^H in HER, indicating that the HEA contains more active sites than single metal catalysts, leading to higher water adsorption and dissociation rate (Figure S32, Supporting Information). The Co site showed the largest Gibbs free energy gap through the water dissociation process (i.e., –0.22 and –2.36 eV, respectively), enabling ^*^H production, which contributes to the overall H_2_ formation on Pt sites and other metal sites. This, in turn, leads to an increase in the total number of active sites and higher H utilization by a wider range of *ΔG*_ad_ to achieve enhancement in HER. Figure 5g and Table S3 (Supporting Information) show a comparison of HER performance of HEA/TiO_2_–NF with recent studies on HEAs and other classes of reference materials. The analysis demonstrated that the PtFeCoNiCu HEA/TiO_2_–NF PEC system outperforms all these forms of catalysts for HER, with a remarkably low overpotential of 11 mV at *η*_10_. The combination of HEA with semiconductors is therefore demonstrated as an optimal approach to designing a new class of highly efficient photoelectrocatalysts. Our analysis suggests that the function of each component in this hybrid system (*i*.*e*., HEA and semiconductor) can be synergistically engineered to enhance HER efficiency. The structure of HEA nanoparticles, controlled by the electrodeposition time, and the crystallographic phase of the semiconductor, which acts as an electronic buffer, are found to be critical aspects to enhancing charge carrier efficiency, and reducing absorption and desorption energy of reactive species involved in HER. However, there remain key fundamental questions to be addressed in terms of the interplay of elements and their distribution in HEAs as well as the characteristics of the semiconductor. These will be addressed in future studies.

## 3 Conclusion

In summary, we reported a facile strategy to fabricate high‐performance hybrid photoelectrocatalysts combining PtFeCoNiCu HEA and TiO_2_–NFs via sequential anodization and electrodeposition. TiO_2_–NFs produced by high voltage anodization of titanium substrates served as both a semiconducting scaffold and nanostructured support for the controlled growth of PtFeCoNiCu HEA nanoparticles via a Volmer–Weber growth mechanism. Structural and compositional analyses revealed that the HEA particles adopted a single‐phase FCC structure and exhibited uniform elemental distribution across the surface, while the TiO_2_–NFs retained their anatase phase, contributing to the photoactivity of the system under illumination and acting as a buffer layer for electrons. Electrochemical characterizations demonstrated a pronounced synergistic enhancement in HER for the HEA/TiO_2_–NF system, outperforming benchmark control systems including bare Ti film, TiO_2_–NF, and HEA‐coated Ti films. The optimized HEA/TiO_2_–NF catalyst achieved a low overpotential of –89 ± 9 mV at η_10_, while showing an additional performance gain under light illumination – highlighting the pivotal role of the semiconductor TiO_2_ in facilitating photogenerated charge separation and transfer. Systematic control over the electrodeposition time revealed that the transition in HEA morphology, from isolated nanoparticles to interconnected island‐like films, critically influenced catalytic efficiency, further demonstrating the pivotal interplay between nanostructure features and photoelectrocatalytic performance. HEA/TiO_2_–NFs synthesized at an optimized electrodeposition time of 120 min exhibited a significantly HER activity with a remarkably low overpotential of –37 mV at *η*_10_, under illumination. Further enhancement was achieved through thermal effect as increasing the electrolyte temperature to 80 °C resulted in an ultralow overpotential of –11 mV at *η*_10_, surpassing most HEA‐based HER catalysts reported in the literature. Notably, the optimized HEA/TiO_2_–NF system also demonstrated excellent operational durability with minimal performance degradation after 10 000 HER cycles at 80 °C, validating its robust stability under industrially relevant high‐temperature and alkaline conditions. We elucidated the HER mechanism of the PtFeCoNiCu HEA/TiO_2_–NF system through a combination of in situ Raman spectroscopy and DFT simulations. The reaction was found to proceed through three distinct stages – non‐reactive, activation, and high‐speed reaction. During the three stages, the TiO_2_ support underwent a transformation, from a passive scaffold to an electron‐buffering layer with significant lattice restructuring. In situ Raman spectral analyses revealed Ti⁴⁺ reduction, hydrogen intercalation, and rapid turnover of interfacial intermediates (OH radicals) under increasing cathodic bias, directly correlating with the enhanced HER kinetics. DFT calculations demonstrated that the PtFeCoNiCu HEA exhibited lower hydrogen adsorption energies than those of anatase TiO_2_, while the anatase TiO_2_ support offered complementary high‐energy adsorption sites. This synergistic interaction broadened the range of accessible active sites and facilitated efficient hydrogen evolution. This study outlines a robust strategy for constructing PtFeCoNiCu HEA/TiO_2_–NF photoelectrocatalysts with exceptional hydrogen evolution activity, achieving an ultralow overpotential of –11 mV at *η*_10_ and excellent thermal stability. The finding of the synergistic interplay between HEA structures and the TiO_2_ support provides valuable mechanistic insights into design principles for next‐generation photoelectrocatalysts.

## 4 Experimental Section

### TiO_2_ Nanofilm Fabrication

TiO_2_ nanofilms were grown on Ti foils (i.e., 0.2 mm thick, Goodfellow, 99.6 + % purity) by anodization. Before anodization, the Ti foils were cut into 1.5 × 1.5 cm chips, washed in EtOH and Milli–Q water, and air dried. Ti foils were anodized at 120 V for 3 min at room temperature in an aqueous 0.5 m H_2_SO_4_ electrolyte (Sigma–Aldrich, Australia).

### Electrodeposition of HEA

Anodic TiO_2_ nanofilms were immersed in the metal precursor aqueous solution containing 2 mm of precursors FeCl_2_∙4H_2_O, NiCl_2_∙6H_2_O, CoCl_2_∙6H_2_O, CuCl_2_ ∙2H_2_O, and H_2_PtCl_6_∙6H_2_O. Electrodeposition was performed in a two‐electrode reactor under a bias of 1 V at room temperature for 40, 60, and 120 min.

### Structural and Chemical Characterization

The morphology and thickness of TiO_2_ nanofilms were characterized by field emission gun scanning electron microscopy (FEG‐SEM Quanta 450, FEI). The thickness of TiO_2_ thin films was determined from FEG‐SEM image analysis using ImageJ and by ellipsometry measurements for validation. The crystal structure of TiO_2_ nanofilms was characterized by X‐ray diffraction (Rigaku SmartLab3), using MDI Jade 9 software to identify lattice type. Raman and electrochemical in situ Raman spectroscopy spectra were acquired in a Raman confocal microscope (LabRAM HR Evolution, Horiba) with laser excitation at 532 nm. HAADF‐STEM and EDX mapping analyses were obtained by a high‐resolution transmission electron (FEI Titan Themis 80–200). The chemical composition profile of HEA/TiO_2_–NFs was obtained by X‐ray photoelectron spectroscopy (XPS, Thermo Fisher Scientific Nexsa). The mass fractions of Pt, Ni, Cu, Co, and Fe in TiO_2_ nanofilms were quantified by inductively coupled plasma mass spectrometry (ICP–MS, Agilent 720ES).

### Photoelectrochemical Measurements

Photoelectrochemical HER measurements were characterized in a three‐electrode system with an aqueous electrolyte of 1 m potassium hydroxide (KOH; pH ≈ 14) saturated with Ar gas, using an electrochemical station (CHI760E, CHI Instruments Inc.). The three‐electrode system consisted of a reference electrode (Hg/HgO), a counter electrode (Pt), and the Ti film, TiO_2_–NF, HEA/Ti film, and HEA/TiO_2_–NF as the working electrodes. The effective area of HEA/TiO_2_–NFs was 0.785 cm^2^ for all the electrochemical measurements. The scan rate of 5 mV s^−1^ was used for all linear sweep voltammetry measurements, from –0.8 to 0.0 V versus RHE. All applied biases were converted into RHE. A 150 W xenon lamp (PLS‐SXE300, Beijing Perfectlight) was used as a light source to simulate broad‐spectrum solar light. Chronoamperometry of the HEA/TiO_2_–NF was performed at ≈0 V versus RHE with 10 s intervals for the ON−OFF illumination modes.

### DFT Computations

All density functional theory (DFT) calculations were conducted by using the Vienna ab‐initio simulation package (VASP)^[^ ^47^ ^]^ with the Perdew‐Burke‐Ernzerhop (PBE)^[^ ^48^ ^]^ exchange‐correlation, and all calculations used the projector‐augmented wave (PAW)^[^ ^49^ ^]^ potential to describe the ionic cores. Anatase and HEA surface structure was calculated by setting a 450‐eV plane‐wave cutoff energy and a Monkhorst‐Pack^[^ ^50^ ^]^ k‐point grid of 3 × 3 × 1. Anatase and HEA surface structures were chosen (001), respectively (Table S4, Supporting Information), whereas Figure S33 (Supporting Information) provides details on the absorption energies the different active sites in the HEA. The ^*^H absorption energy of varied active sites on the PtFeCoNiCu HEA revealed that Pt exhibited the strongest adsorption among these sites with a value of –0.68 eV, whereas the Fe site showed the weakest adsorption energy of 1.79 eV. The presence of multiple sites from high entropy alloys led to the increasing absorption rate and amount of ^*^H, which resulted in a higher reaction rate and hydrogen production. The high‐entropy alloy was generated using the ATAT programme by constructing an FCC structure, then forming a 3 × 3 × 3 supercell containing 108 atoms (Figure S34, Supporting Information). The model the FCC crystal cell sizes for Co, Cu, Fe, Pt, and Ni atoms was built at a composition of 0.028, 0.111, 0.213, 0.592, and 0.056, respectively. Subsequently, the most disordered structure was identified. The cell size of the HEA was then defined proportionally, based on the pure metal FCC crystal cell size (i.e., 3.81 Å). The structure underwent stretching and scaling via DFT calculations, demonstrating the original model structure as the most stable configuration as it containthe combionaed the lowest total energy.^[^ ^51^ ^]^ The DFT‐D3^[^ ^52^ ^]^ was utilized to address the Van der Waals interactions, the convergence of the energy was set to be 1 × 10^−4^ eV, and that of geometry optimization was set to be maximum force ≤ 0.03 eV A^−1^. The 3d orbital electrons of Ti were treated using the GGA+U method of Dudarev et al. with an effective Hubbard field Coulomb interaction parameter (*U*’ = *U* – *J*).^[^ ^53^ ^]^ According to the literature, the choice of *U* = 4 eV for the surface structure of rutile and anatase types was confident.^[^ ^54^ ^]^ The spin polarization calculations were conducted on the HEA surface structures containing Fe, Co, and Ni, each with initial magnetic moments of 3, 2, and 1 µB, respectively. The adsorption free energy (*G*_ad_) of hydrogen at the TiO_2_ and HEA is calculated as:^[^ ^55^ ^]^

(1) $$G_{ad}=E_{ad}+ZPE+\int C_{P}dT-TS$$

where *E*_ad_ is the adsorption energy, ZPE the zero‐point energy, $\int C_{P}dT$ the enthalpic temperature correction, and TS the entropic correction.

The adsorption energy (*E*_ad_) is calculated as:

(2) $$E_{ad}=E_{Tot}-E_{Surf}-\frac{1}{2}\mu_{H_{2}}$$

where *E*_Tot_ is the DFT total energy of the surface with adsorbate, *E*_Surf_ is the energy of the corresponding pristine surface, and $\mu_{H_{2}}$ is the chemical potential for H_2_ (6.78 eV).

### Statistical Analysis

All the statistics were performed in OriginPro 2025. All data are presented as mean ± SD from an average of measurements of at least 3 samples.

## Conflict of Interest

The authors declare no conflict of interest.

## Supporting information



Supporting Information

## Data Availability

The data that support the findings of this study are available from the corresponding author upon reasonable request.
