# Supplementary material for: Coupling of Engineered High Entropy Alloys with Semiconducting TiO2 Nanofilms for Scalable and Ultrafast Alkaline Hydrogen Evolution Reaction
Source: Adv Sci (Weinh). 2025 Oct 15;13(2):e14558. doi: 10.1002/advs.202514558 (PMC12786308; doi:10.1002/advs.202514558)
Supplement: Supplementary file 1 — Supporting Information [file ADVS-13-e14558-s001.docx]

Supporting Information

**Coupling of Engineered High Entropy Alloys with Semiconducting TiO_2_ Nanofilms for Scalable and Ultrafast Alkaline Hydrogen Evolution Reaction**

Zichu Zhao, Yanzhang Zhao, Wenqiang Wang, Xiaying Xin, Yan Jiao, Andrew D. Abell, Cheryl Suwen Law^*^, and Abel Santos^*^


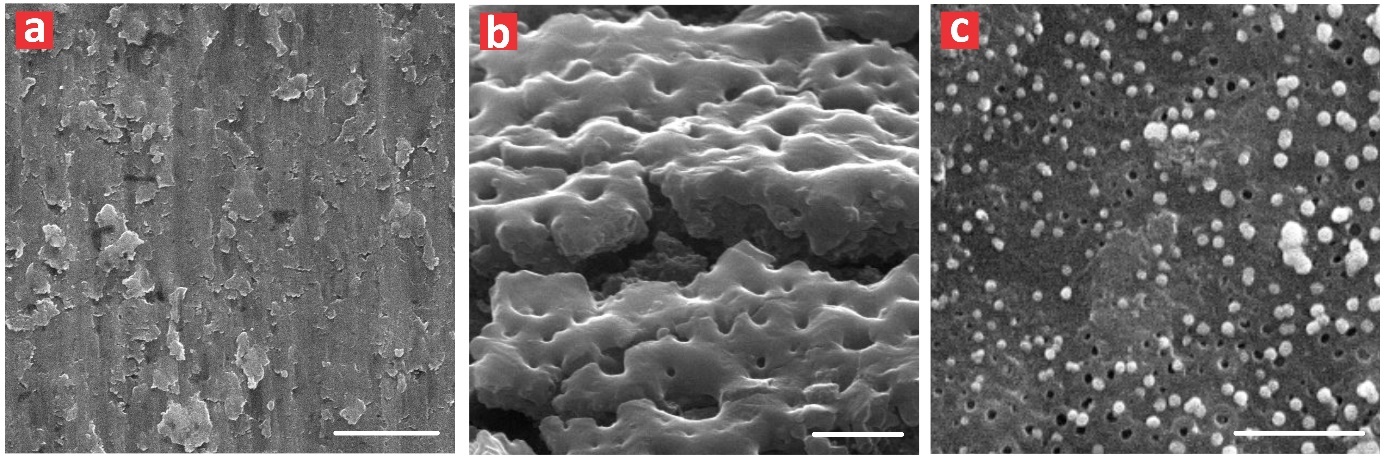


**Figure S1.** Top view of FEG-SEM images of (a) Ti film, (b) TiO_2_–NF and (c) HEA/TiO_2_–NF, Ti film is unmodified, TiO_2_–NF fabricated at 120 V and HEA/TiO_2_–NF produced by anodization at 120 V and electrodeposition at 1 V for 40 minutes.


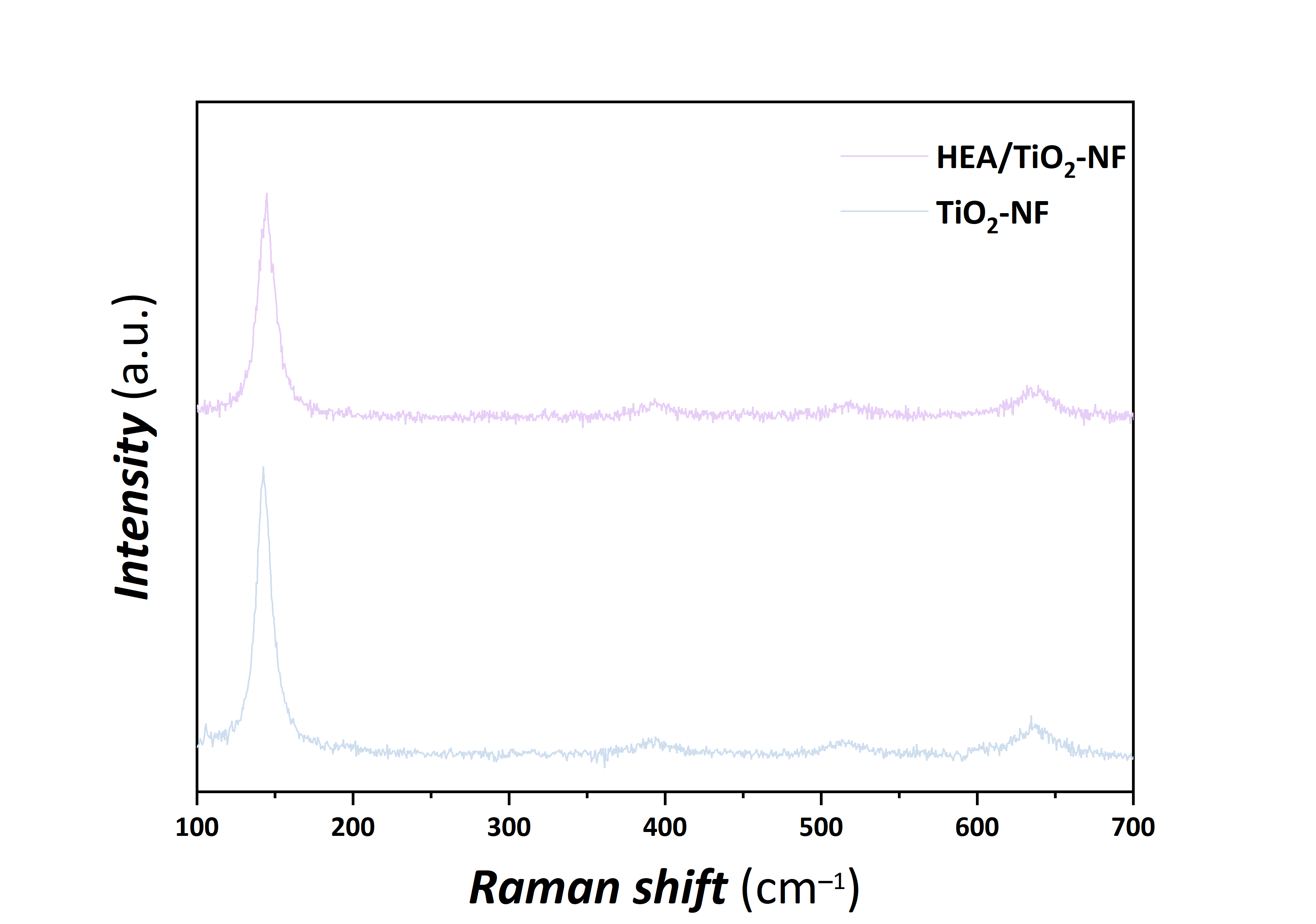


**Figure S2.** Raman spectrum of representative TiO_2_–NF and HEA/TiO_2_–NF produced by anodization at 120 V, and anodization at 120 V and electrodeposition at 1 V for 40 minutes, respectively.


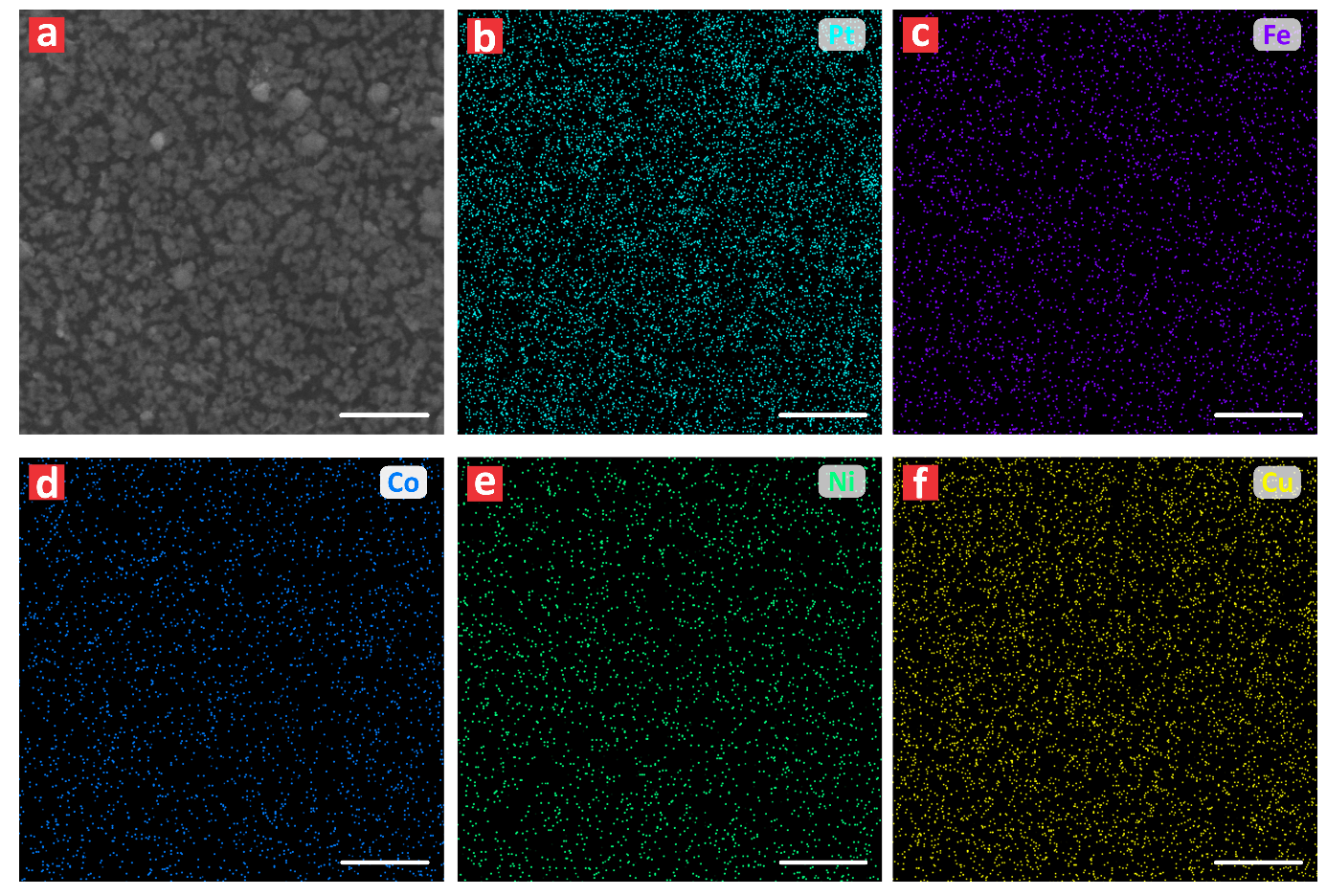


**Figure S3.** (a) Top view of FEG-SEM images of a representative HEA/TiO_2_–NF produced by anodization at 120 V and electrodeposition at 1 V for 120 minutes, (b–f) relative EDX mapping of Pt, Fe, Co, Ni, and Cu elements.


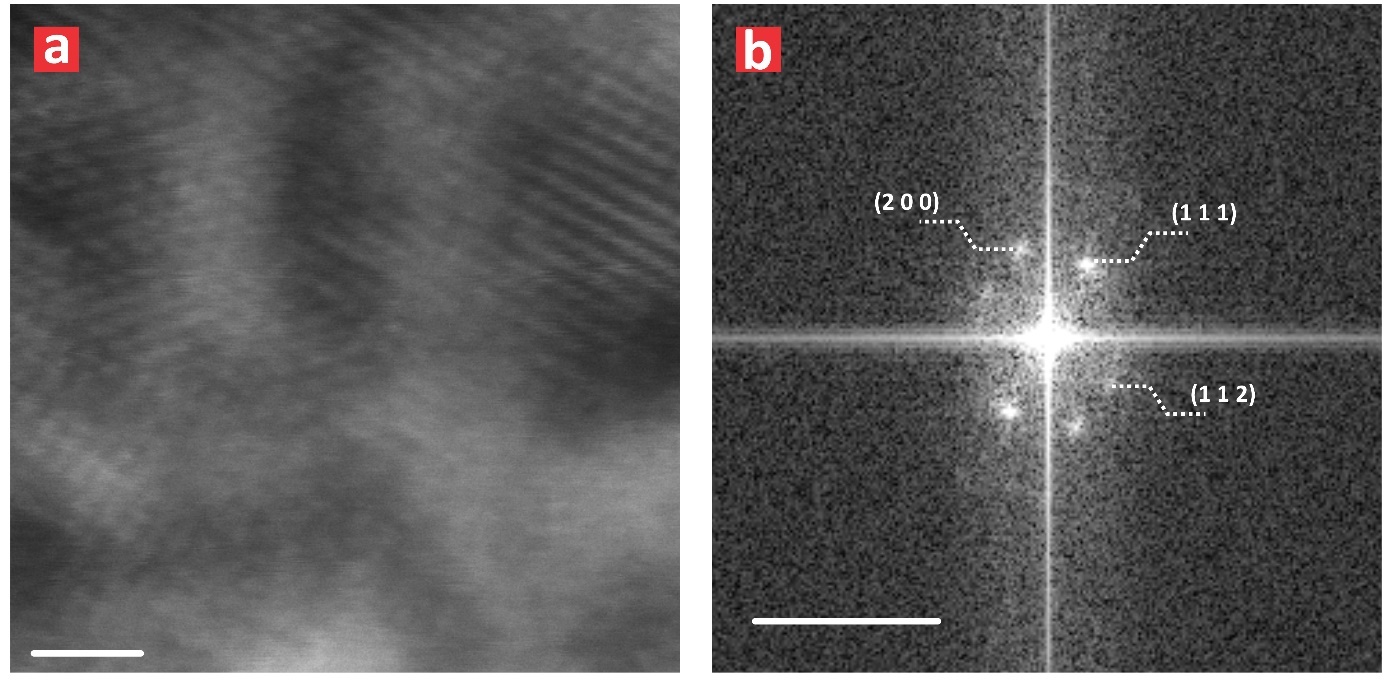


**Figure S4.** (a) HAADF-TEM images of a PtFeCoNiCu HEA/TiO_2_–NF, (b) corresponding FFT patterns of (a) to further verify the structural phases of the monolithic PEC system (scale bars = 5 nm and 5 nm^–1^, respectively).


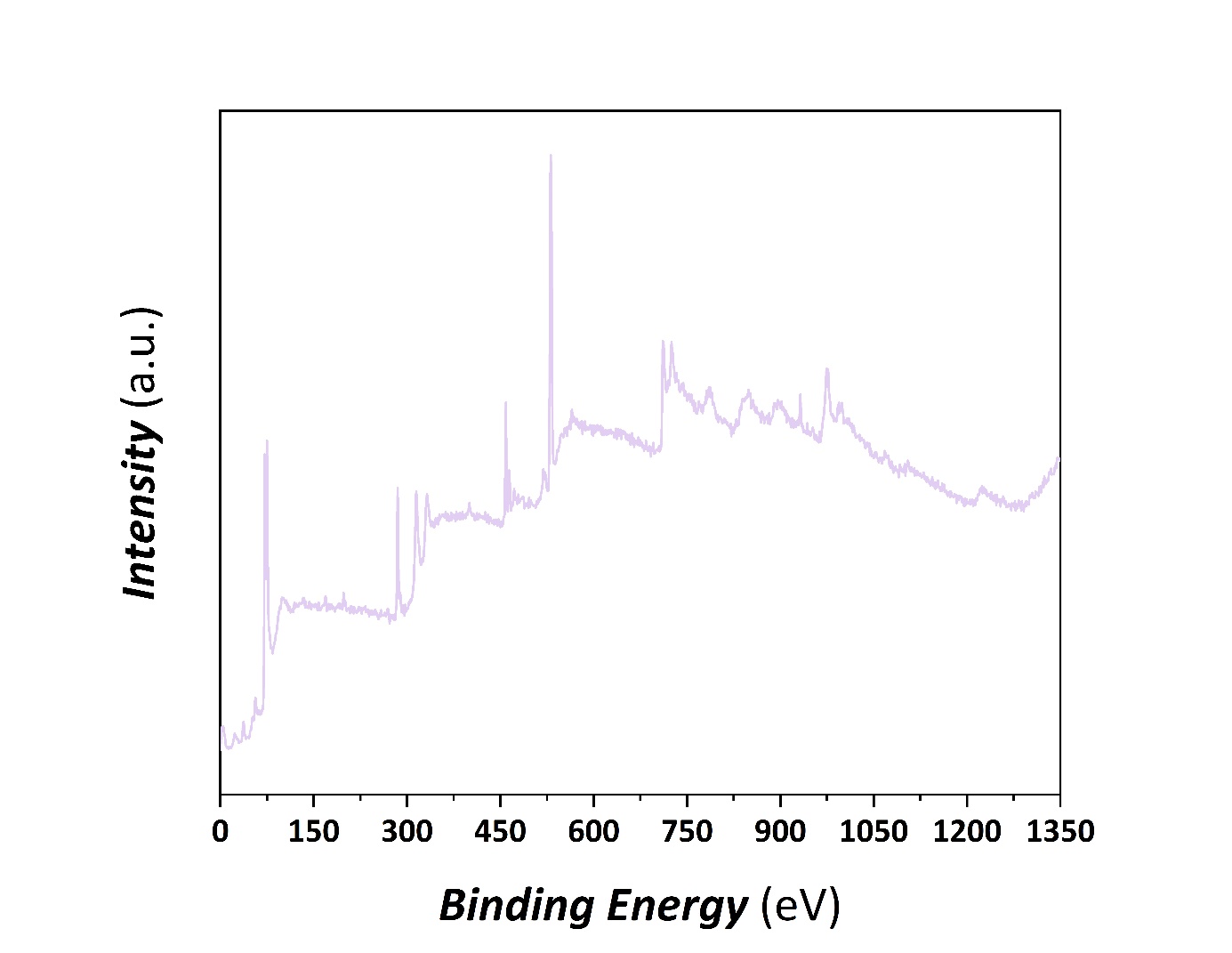


**Figure S5.** High-resolution XPS full spectra of a representative PtFeCoNiCu HEA/TiO2–NF system.


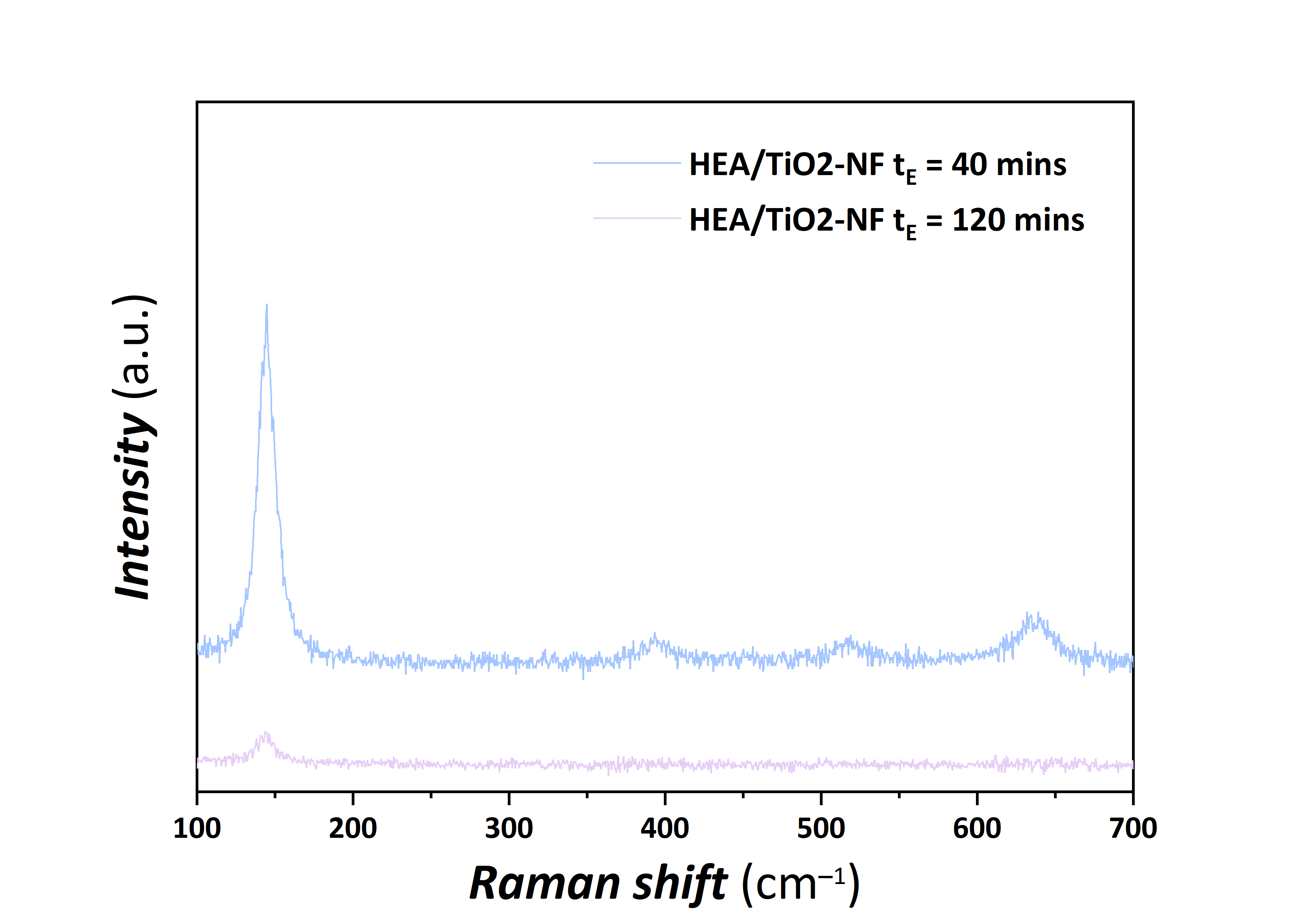


**Figure S6.** Raman spectra of a representative HEA/TiO_2_–NF fabricated at 120 V and distinct electrodeposition time at 1 V for 40 and 120 minutes.


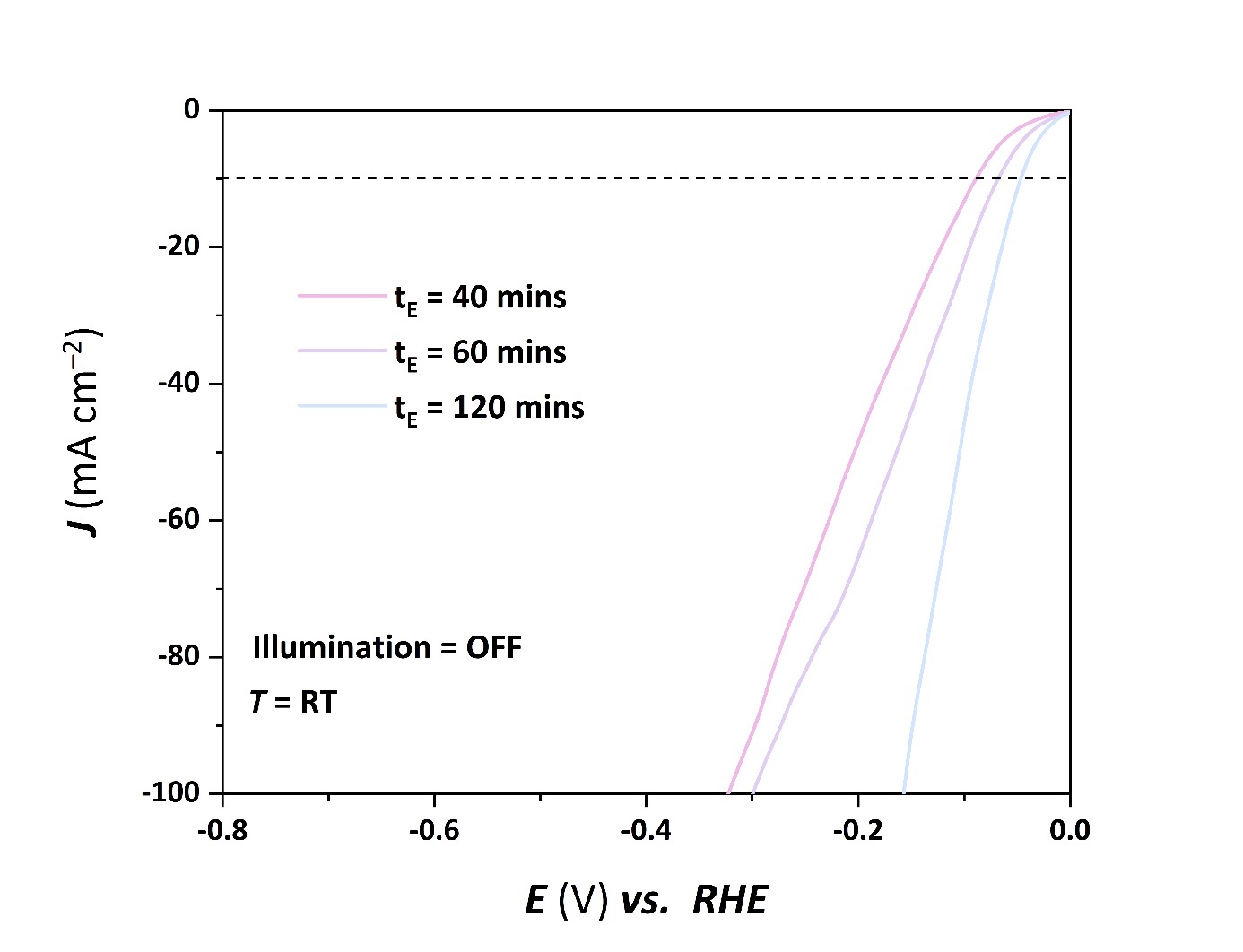


**Figure S7.** Linear sweep voltammograms of PtFeCoNiCu HEA/TiO_2_–NFs synthesized at electrodeposition times of 40, 60 and 120 minutes under varying overpotential (*E*), from –0.8 to 0.0 V *vs* RHE, at a rate of 0.005 V s^–1^ in 1 M KOH.


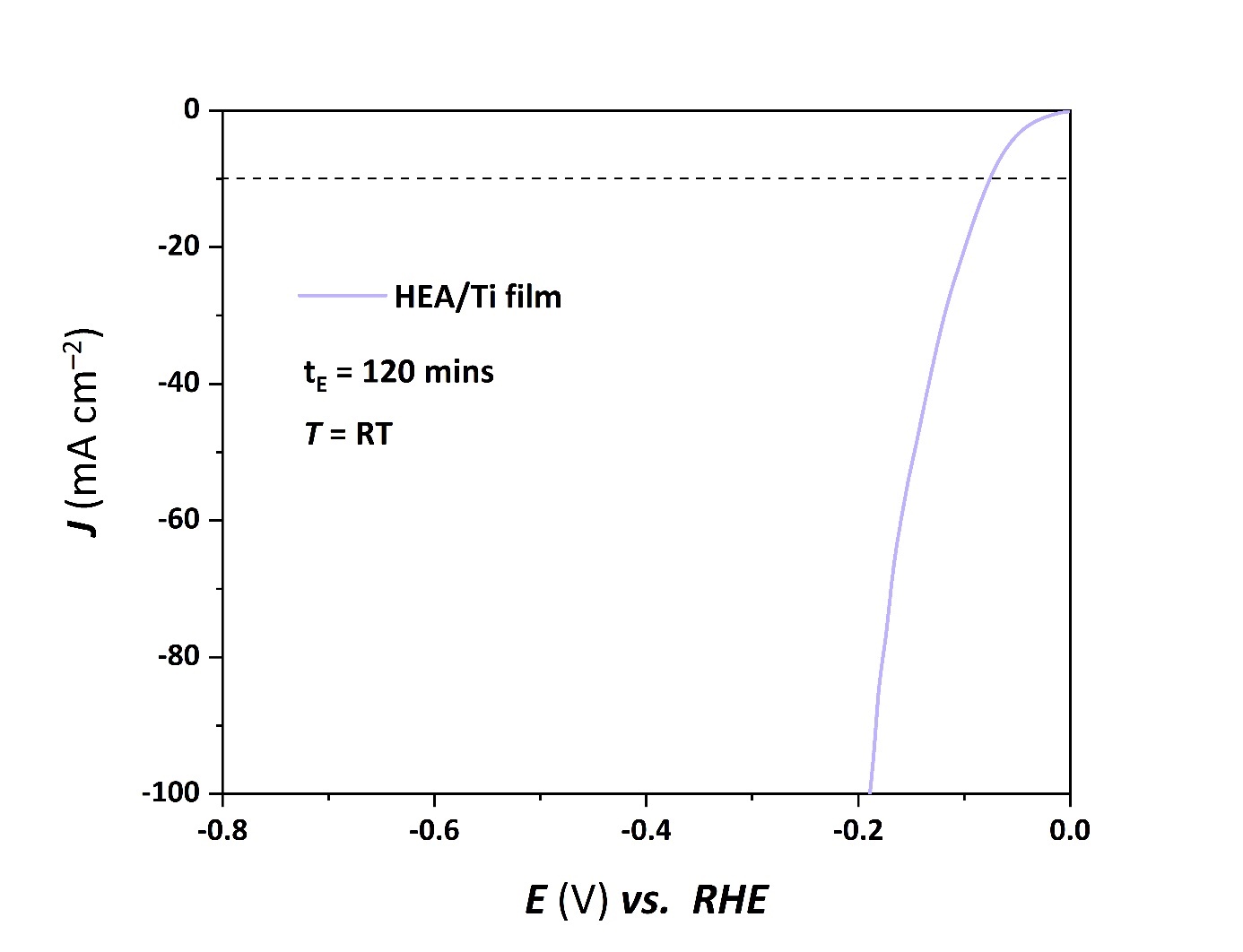


**Figure S8.** Linear sweep voltammograms of PtFeCoNiCu HEA/Ti film synthesized at electrodeposition times of 120 minutes under varying overpotential (*E*), from –0.8 to 0.0 V *vs* RHE, at a rate of 0.005 V s^–1^ in 1 M KOH.


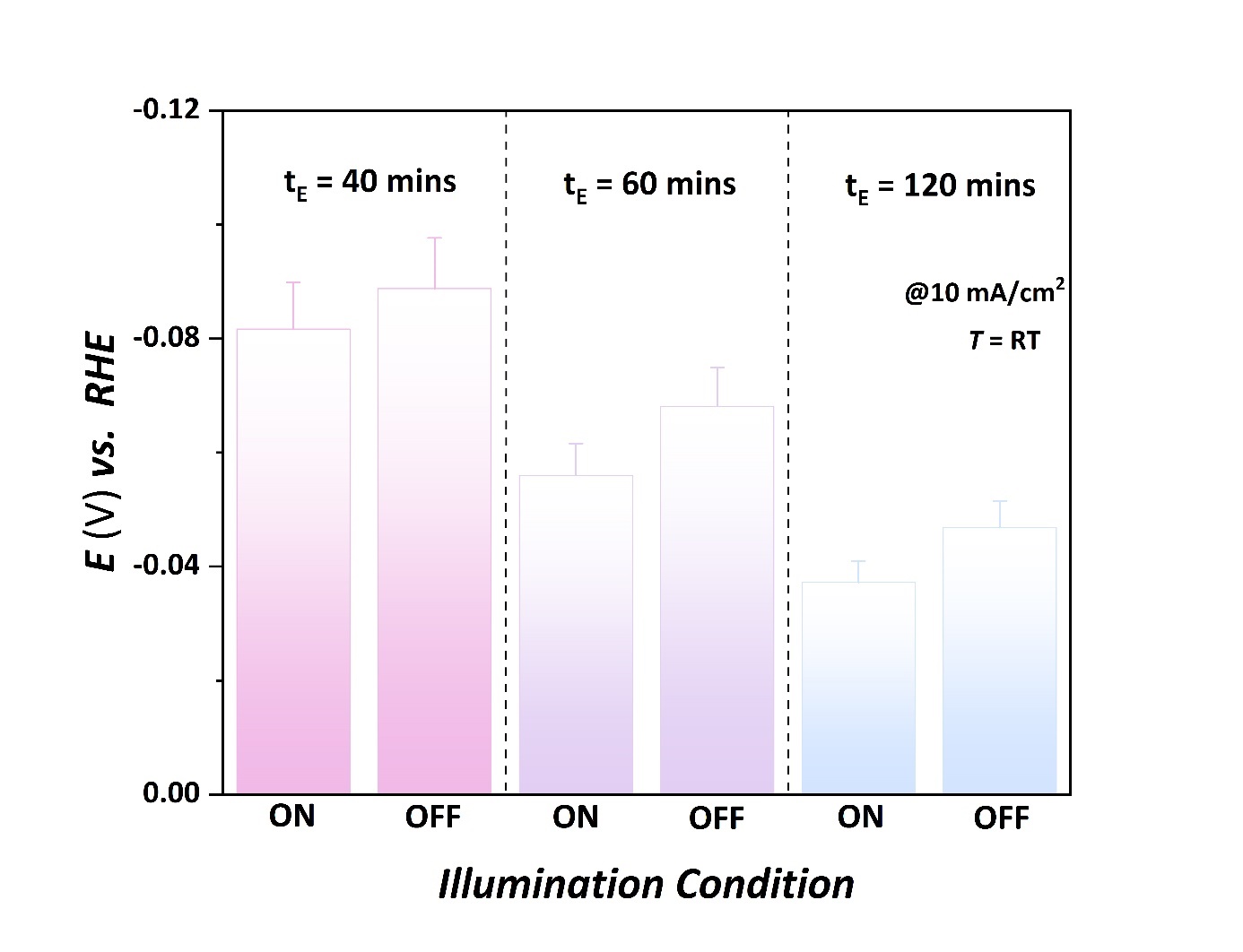


**Figure S9.** Summary of overpotential values *vs* RHE at *η*_10_ of PtFeCoNiCu HEA/TiO_2_–NFs synthesized at electrodeposition times of 40, 60 and 120 minutess under ON and OFF illumination states.


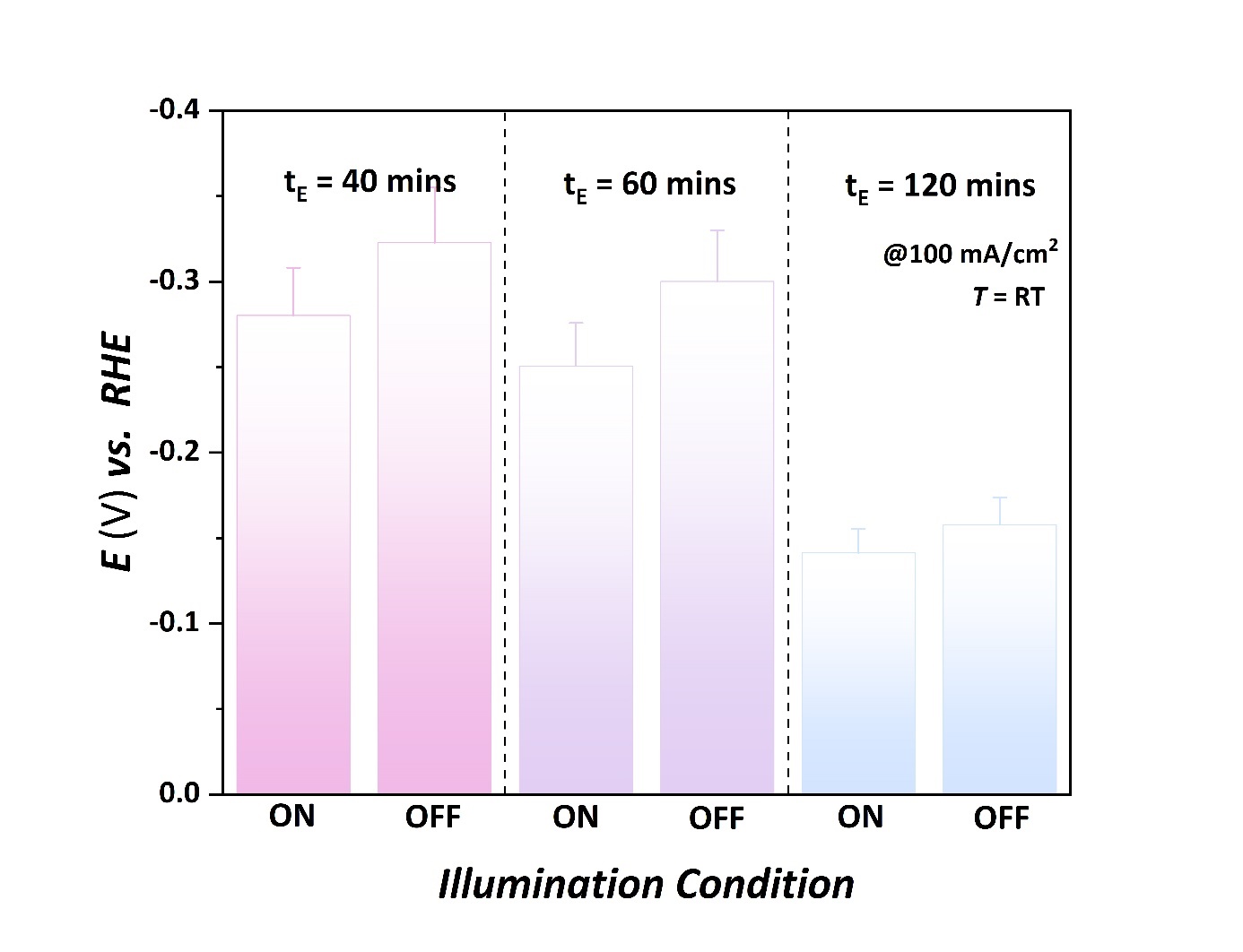


**Figure S10.** Summary of overpotential values *vs* RHE at *η*_100_ of PtFeCoNiCu HEA/TiO_2_–NFs synthesized at electrodeposition times of 40, 60 and 120 minutess under ON and OFF illumination states.


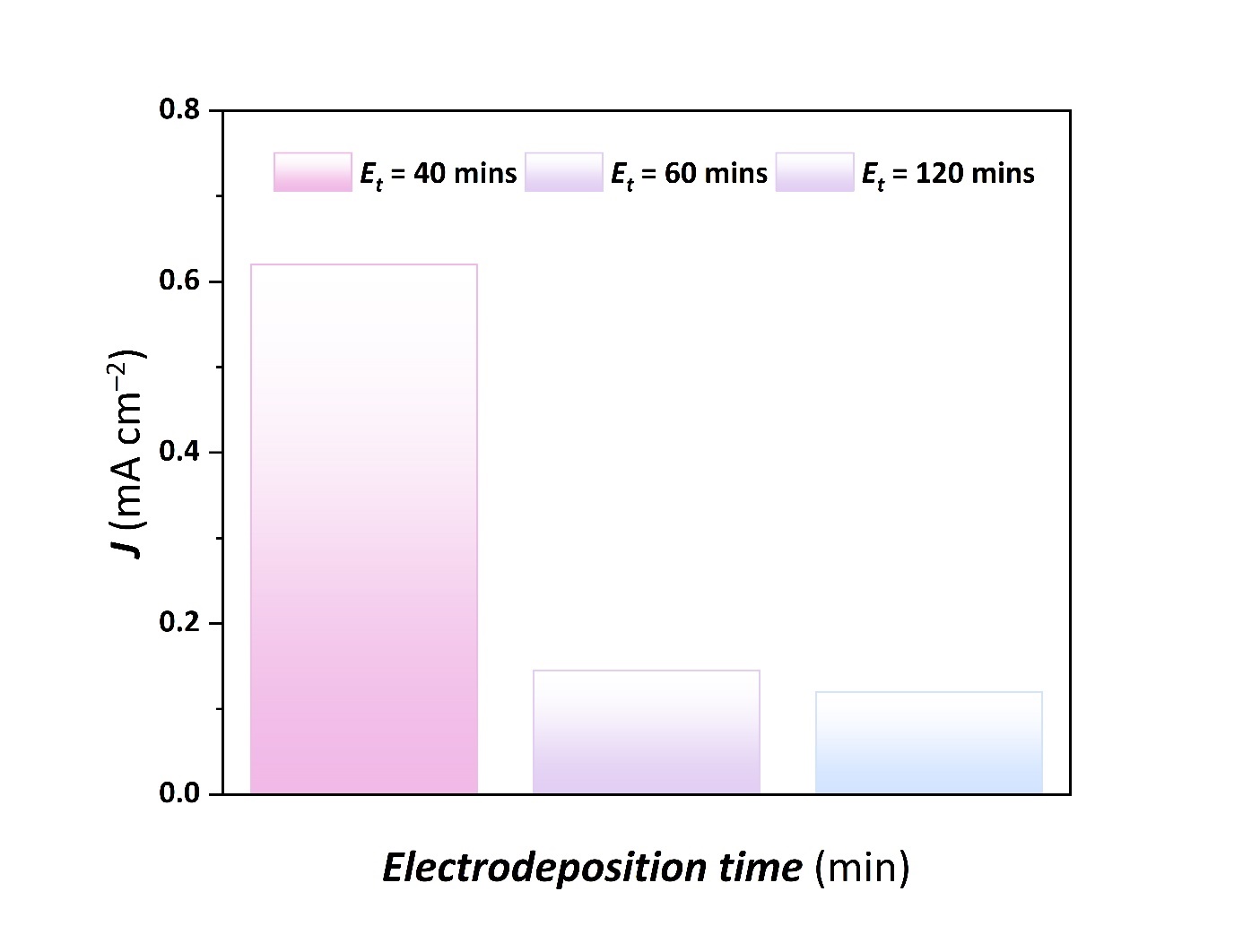


**Figure S11.** Summary of current density values at 0 V *vs* RHE of PtFeCoNiCu HEA/TiO_2_–NFs synthesized at electrodeposition times of 40, 60 and 120 mins under ON and OFF illumation states with 10 seconds intervals.


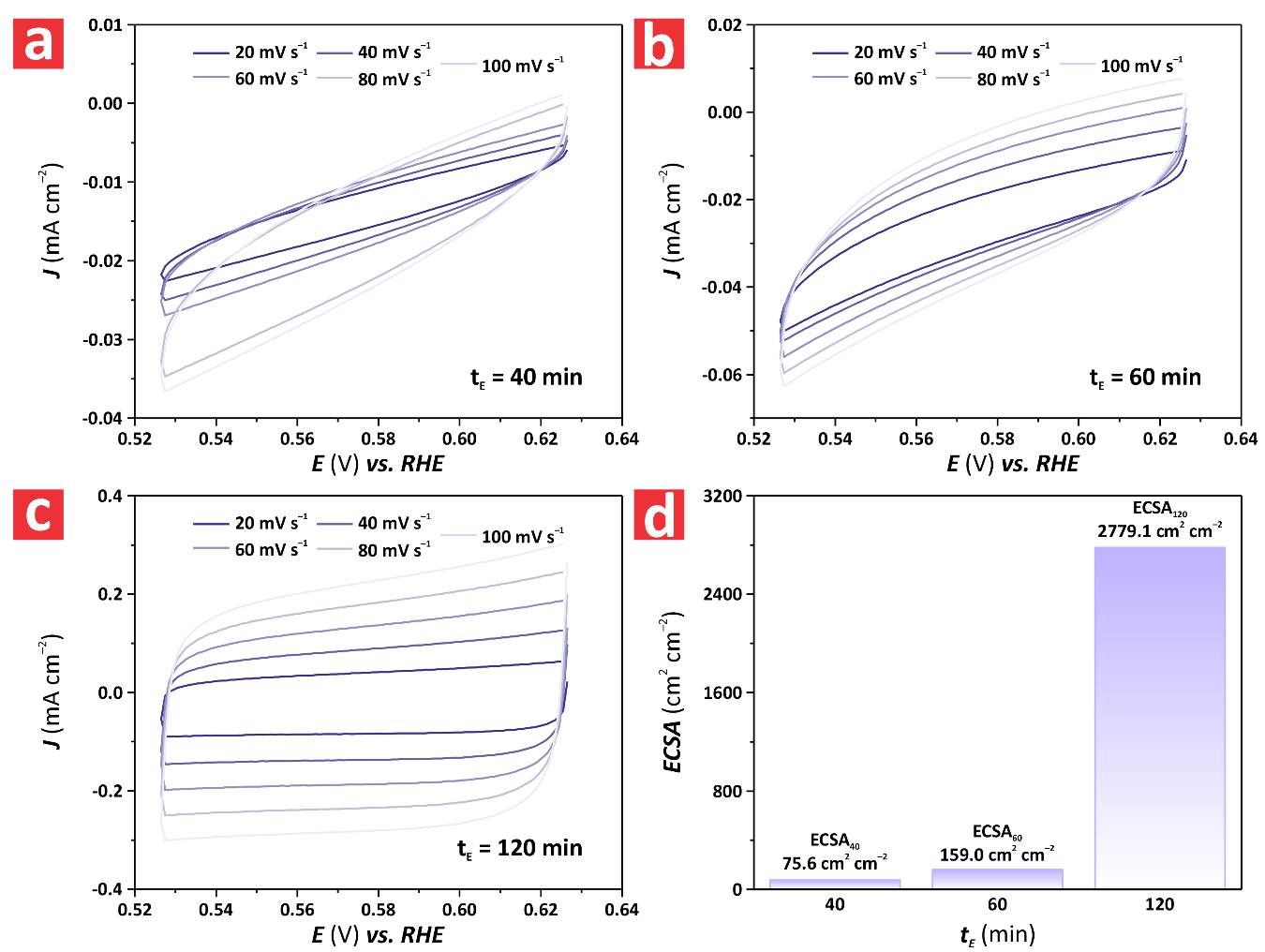


**Figure S12.** Cyclic voltammetry, estimation of ammetry plots and the electrochemical surface area (ECSA) of PtFeCoNiCu HEA/TiO_2_–NFs fabricated at electrodeposition times of 40, 60 and 120 minutes. (a) Cyclic voltammetry graphs of a PtFeCoNiCu HEA/TiO_2_–NF fabricated at *t*_E_ = 40 minutes. (b) Cyclic voltammetry graphs of a PtFeCoNiCu HEA/TiO_2_–NF fabricated at *t*_E_ = 60 minutes. (d) Cyclic voltammetry graphs of a PtFeCoNiCu HEA/TiO_2_–NF fabricated at *t*_E_ = 120 minutes. (d) Electrochemical surface area of PtFeCoNiCu HEA/TiO_2_–NFs fabricated at electrodeposition times of 40, 60 and 120 minutes. (NB 1: data are presented as mean ± SD of a sample size of n ≥ 3 independent measurements; NB 2: ECSA values were calculated by this using the equation: ECSA = *C*_dl_ / *C*_s_, where *C*_dl_ is the double-layer capacitance extracted from the cyclic voltammetry graphs and *C*_s_ is the specific capacitance (*i.e.*, *C*_s_ = 0.86 µF cm^–2^ as previously reported [1]).


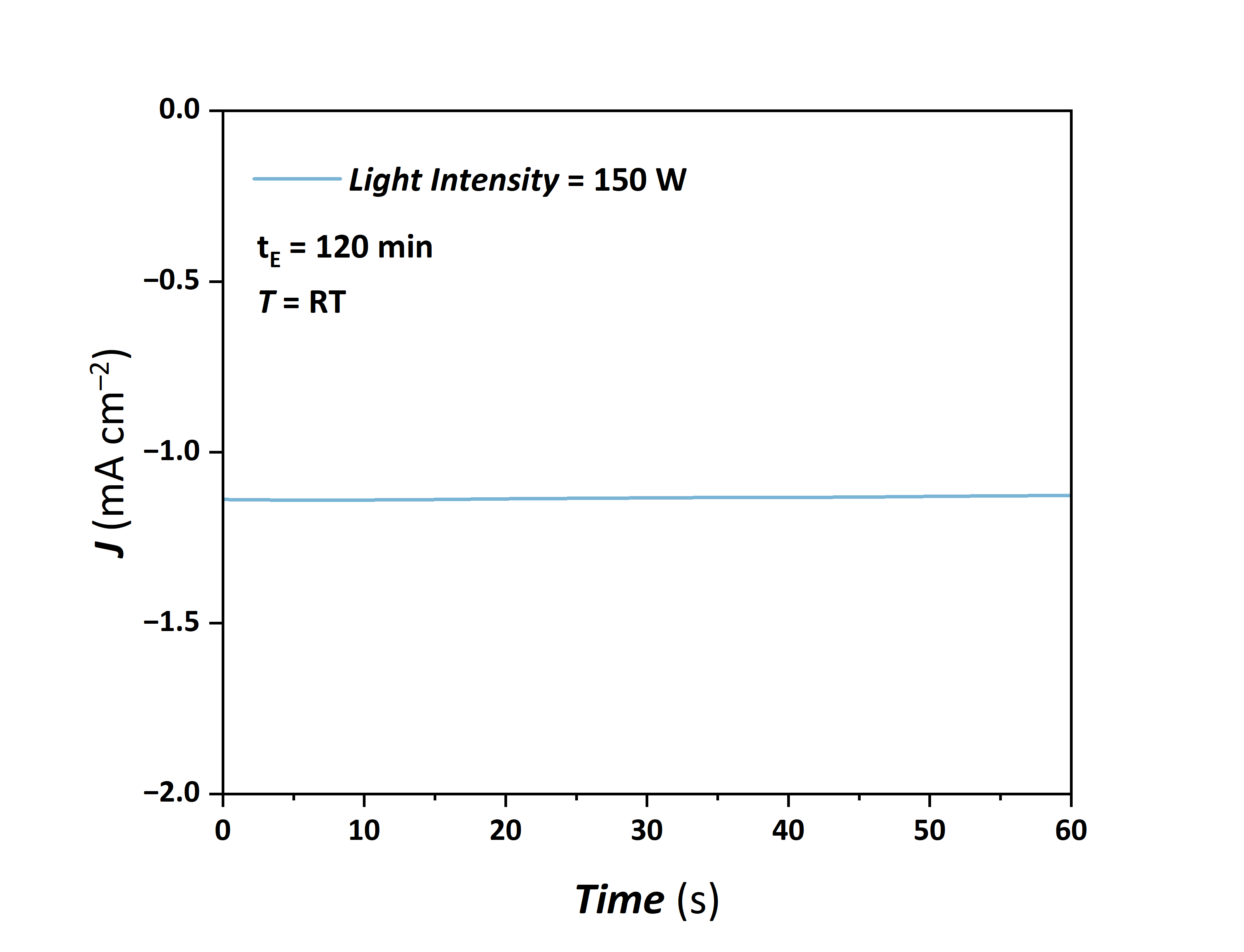


**Figure S13.** Current density under no external bias and ON illumination (*i.e.*, photocatalysis) at a light intensity of 150 W for the PtFeCoNiCu HEA/TiO_2_–NF system synthesized at an electrodeposition time of 120 minutes.


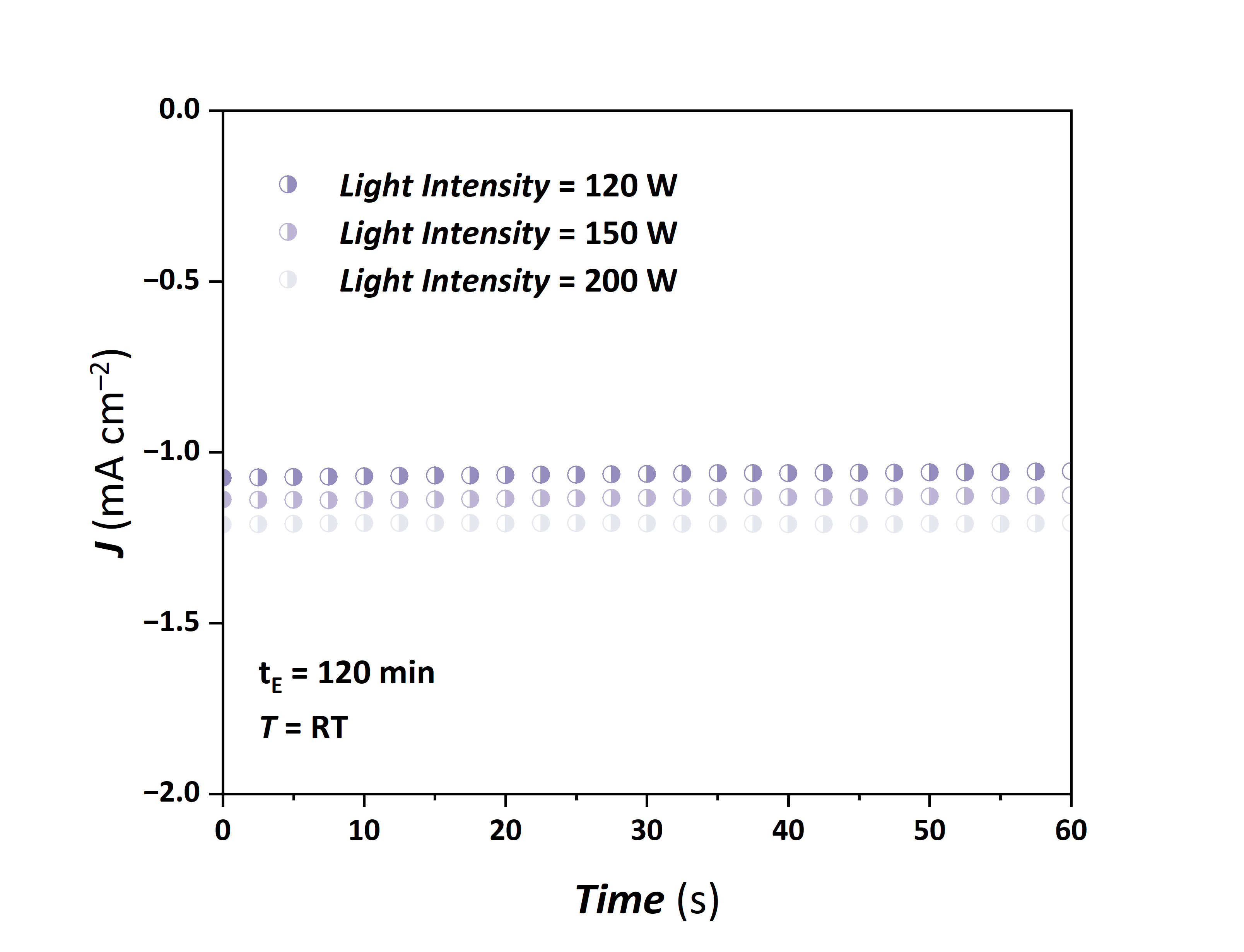


**Figure S14.** Current density under no external bias and ON illumination (*i.e.*, photocatalysis) at varying light intensity, from 120 to 200 W for the PtFeCoNiCu HEA/TiO_2_–NF system synthesized at an electrodeposition time of 120 minutes.


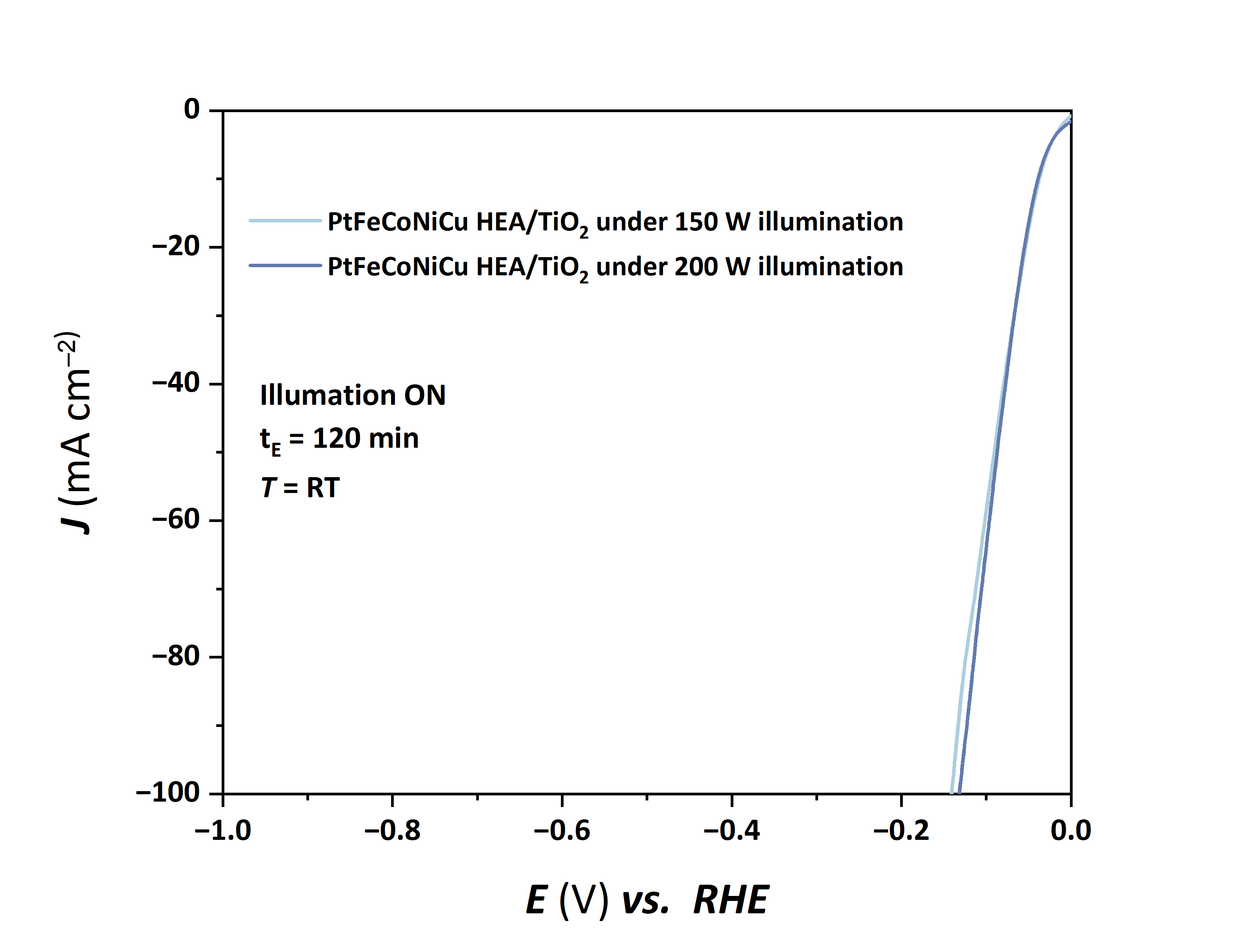


**Figure S15.** Linear sweep voltammograms of the PtFeCoNiCu HEA/TiO_2_–NF system synthesized at an electrodeposition time of 120 minutes under varying overpotential (*E*), from –0.8 to 0.0 V *vs* RHE, at a rate of 0.005 V s^–1^ in 1 M KOH, and illumination intensities of 150 and 200 W.


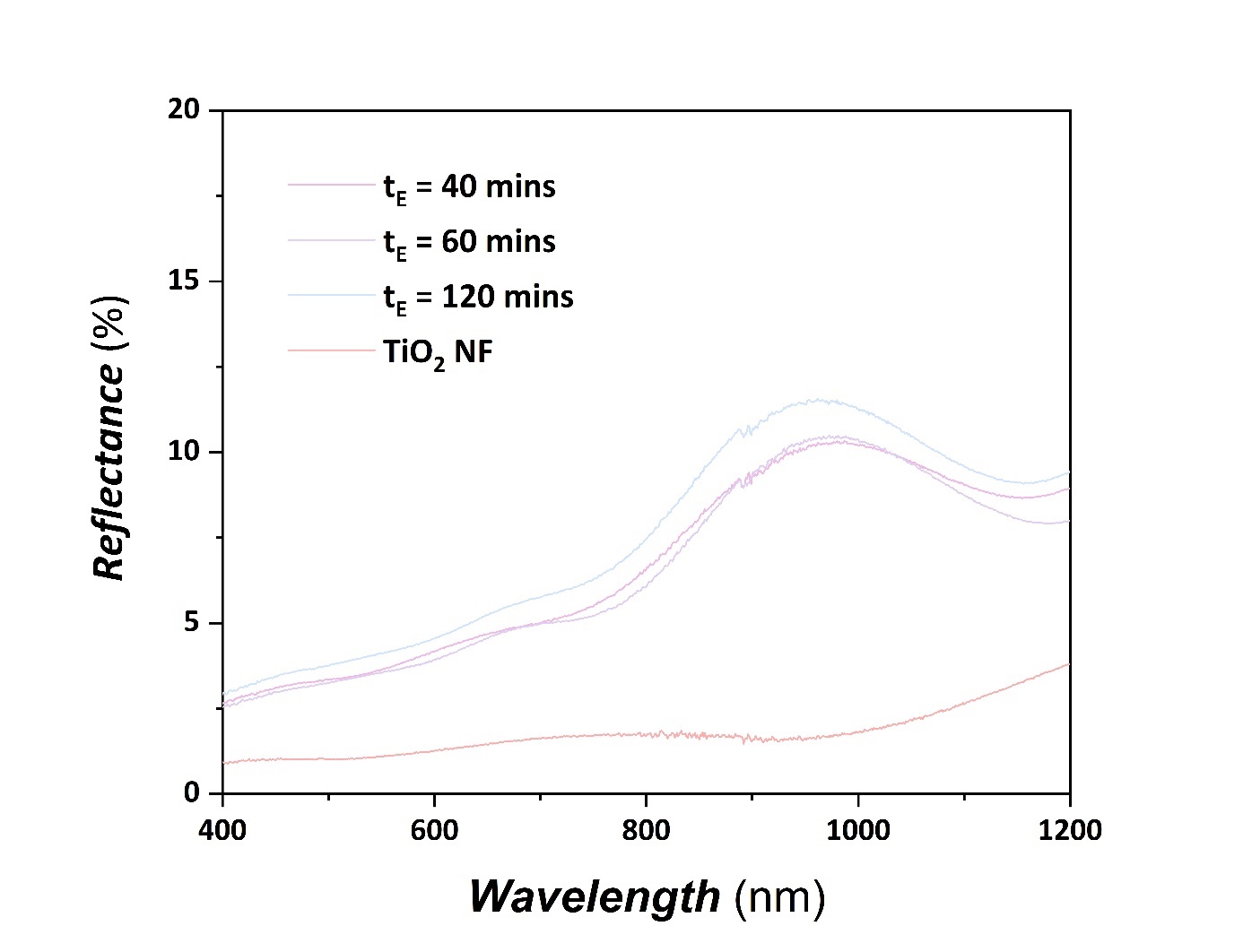


**Figure S16.** UV–visible–NIR reflectance specta of PtFeCoNiCu HEA/TiO_2_–NFs synthesized by anodization at 120 V and electrodeposition at 1 V for 40, 60 and 120 minutes and a reference TiO_2_–NF.


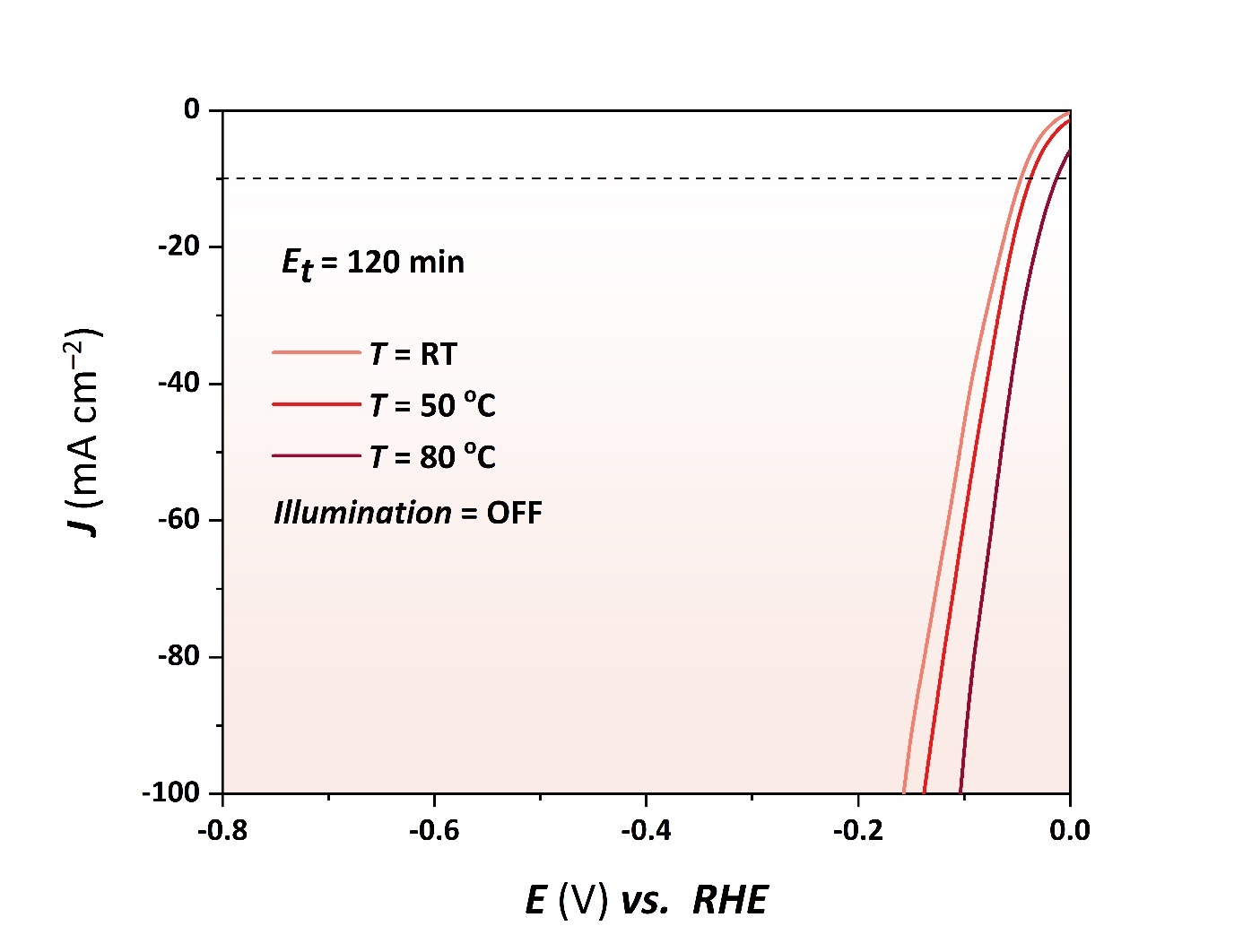


**Figure S17.** Linear sweep voltammograms of the PtFeCoNiCu HEA/TiO_2_–NF synthesized by anodization at 120 V and electrodeposition at 1 V for 120 minutes at *T*_R_ = RT, 50 and 80 °C under OFF illumination state and varying overpotential (*E*), from –0.8 to 0.0 V *vs* RHE at a rate of 0.005 V s^–1^ in 1 M KOH.


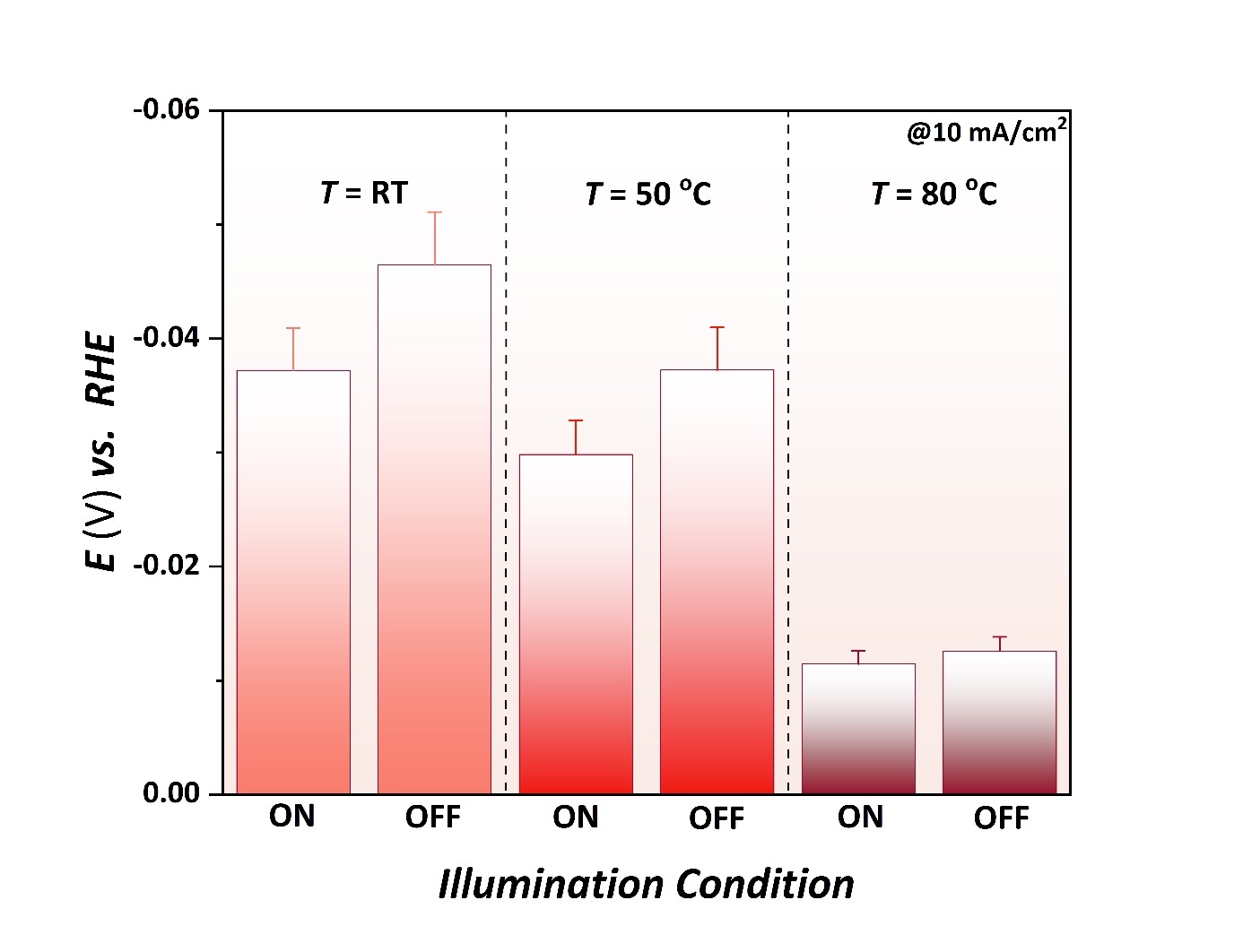
**Figure S18.** Summary of overpotential values *vs* RHE at *η*_10_ for PtFeCoNiCu HEA/TiO_2_–NFs synthesized by anodization at 120 V and electrodeposition at 1 V for 120 minutes at *T*_R_ = RT, 50 and 80 °C under ON and OFF illumination states.


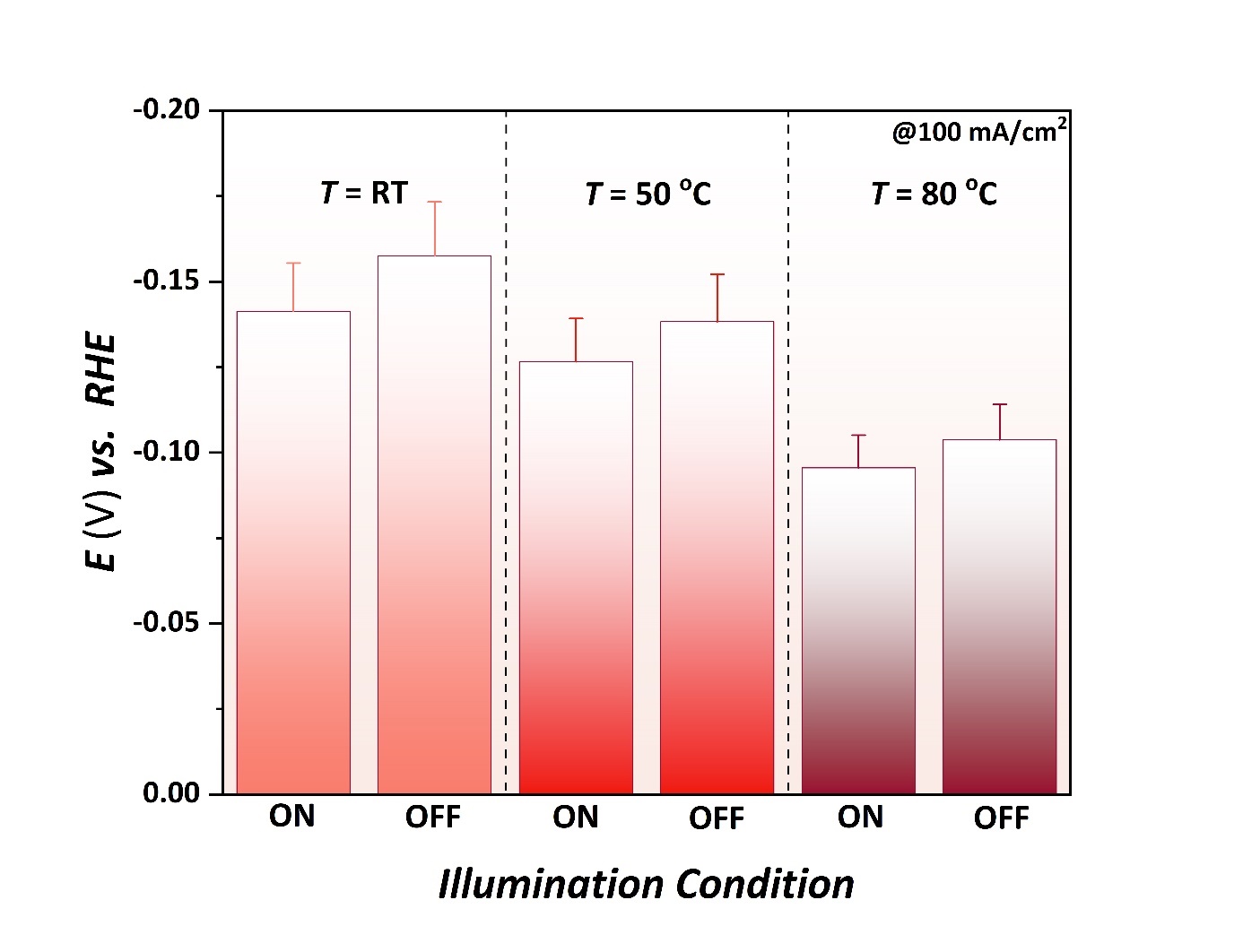


**Figure S19.** Summary of overpotential values *vs* RHE at *η*_100_ of PtFeCoNiCu HEA/TiO_2_–NFs synthesized by anodization at 120 V and electrodeposition at 1 V for 120 minutes at *T*_R_ = RT, 50 and 80 °C under ON and OFF illumination states.


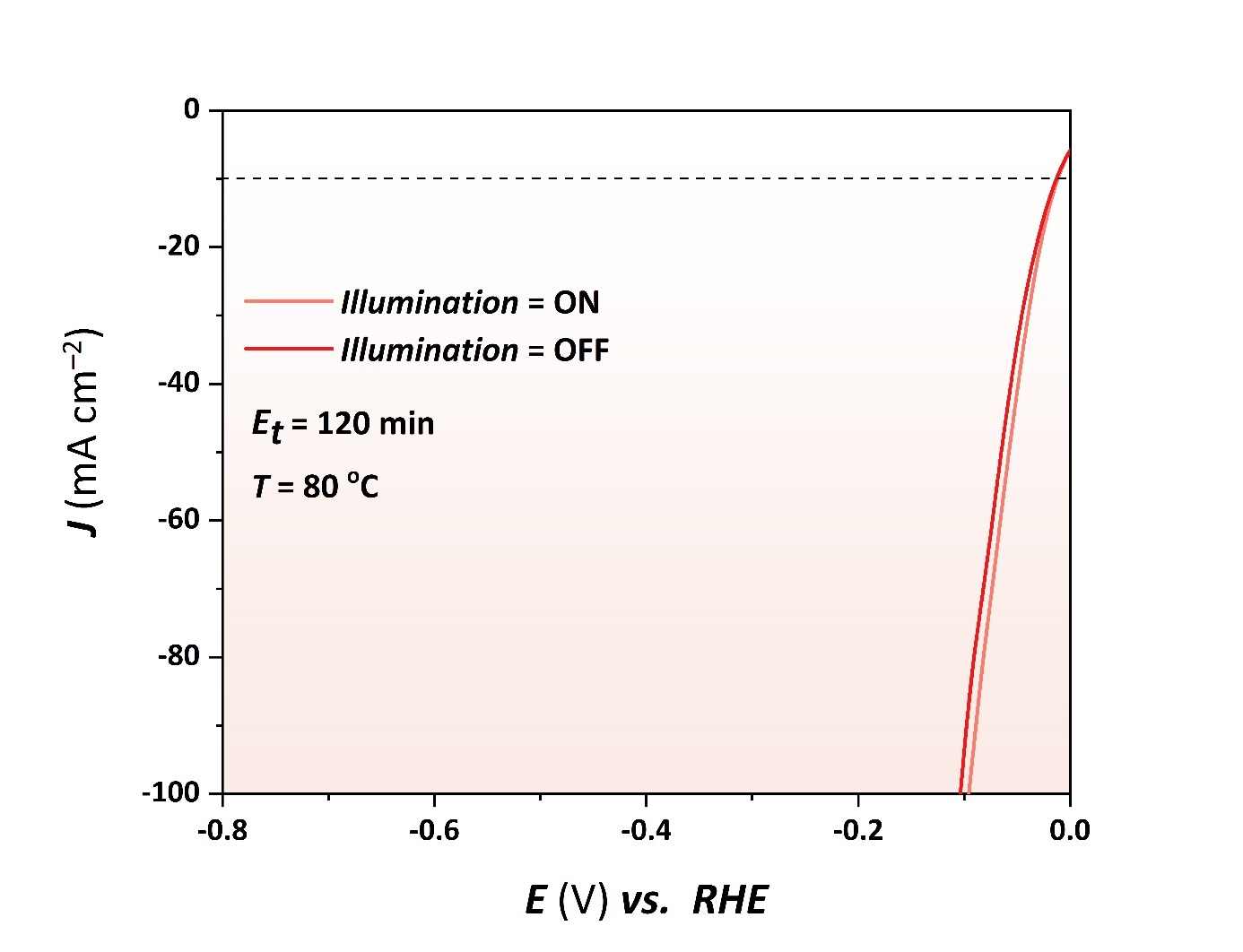


**Figure S20.** Linear sweep voltammograms of the PtFeCoNiCu HEA/TiO_2_–NF synthesized by anodization at 120 V and electrodeposition at 1 V for 120 minutes at *T*_R_ = RT, 50 and 80 °C under ON and OFF illumination states and varying overpotential (*E*), from –0.8 to 0.0 V *vs* RHE at a rate of 0.005 V s^–1^ in 1 M KOH.


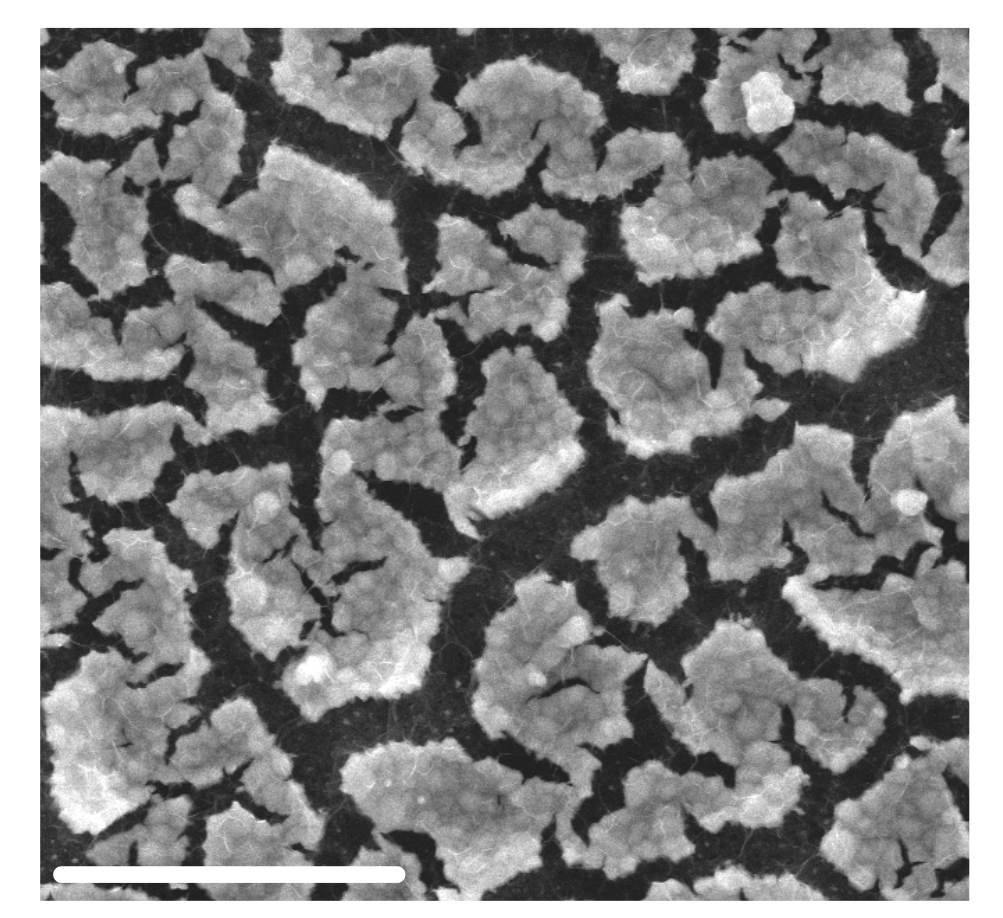


**Figure S21.** Top view of FEG-SEM images of a HEA/TiO_2_–NF, Ti film produced by anodization at 120 V and electrodeposition at 1 V for 120 minutes after 5000 HER cycles at an electrolyte temperature of 80 °C (scale bar = 10 μm).


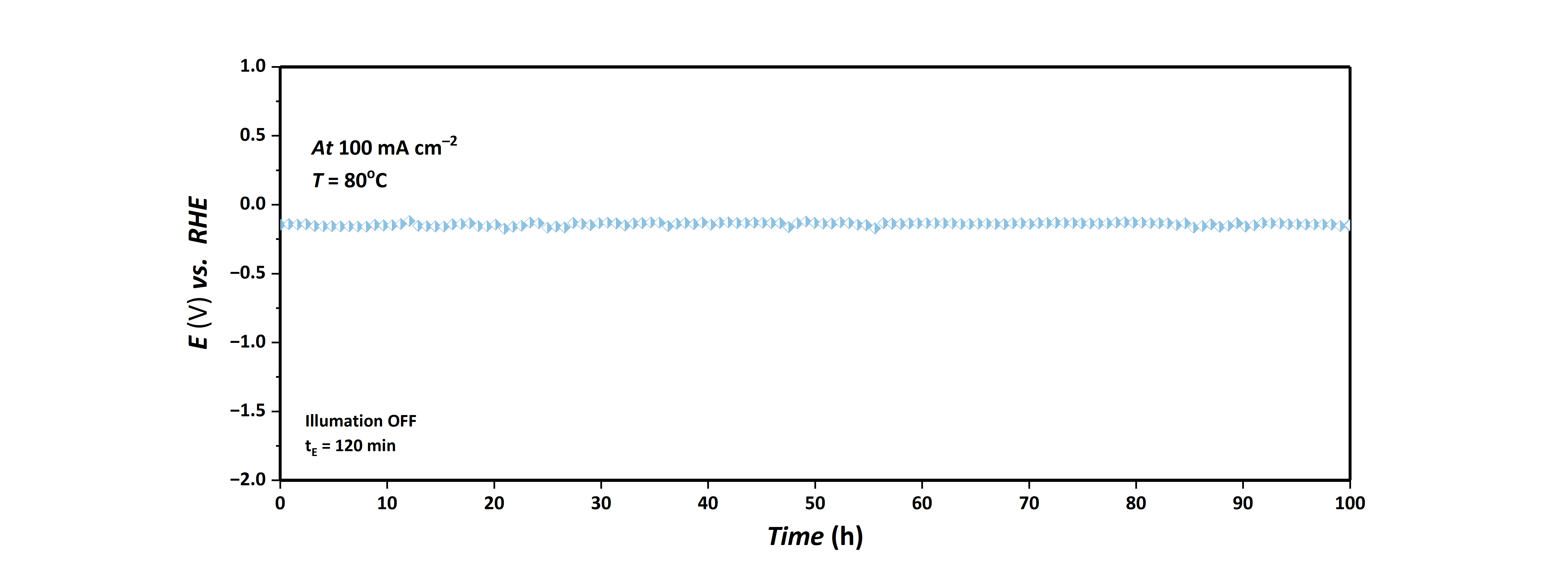


**Figure S22.** Long-term stability test of the PtFeCoNiCu HEA/TiO_2_–NFs system synthesized at an electrodeposition time of 120 minutes at an input current dentisy of 100 mA cm^–2^ under OFF illumination state and 80 °C electrolyte operating temperature in 1M KOH.


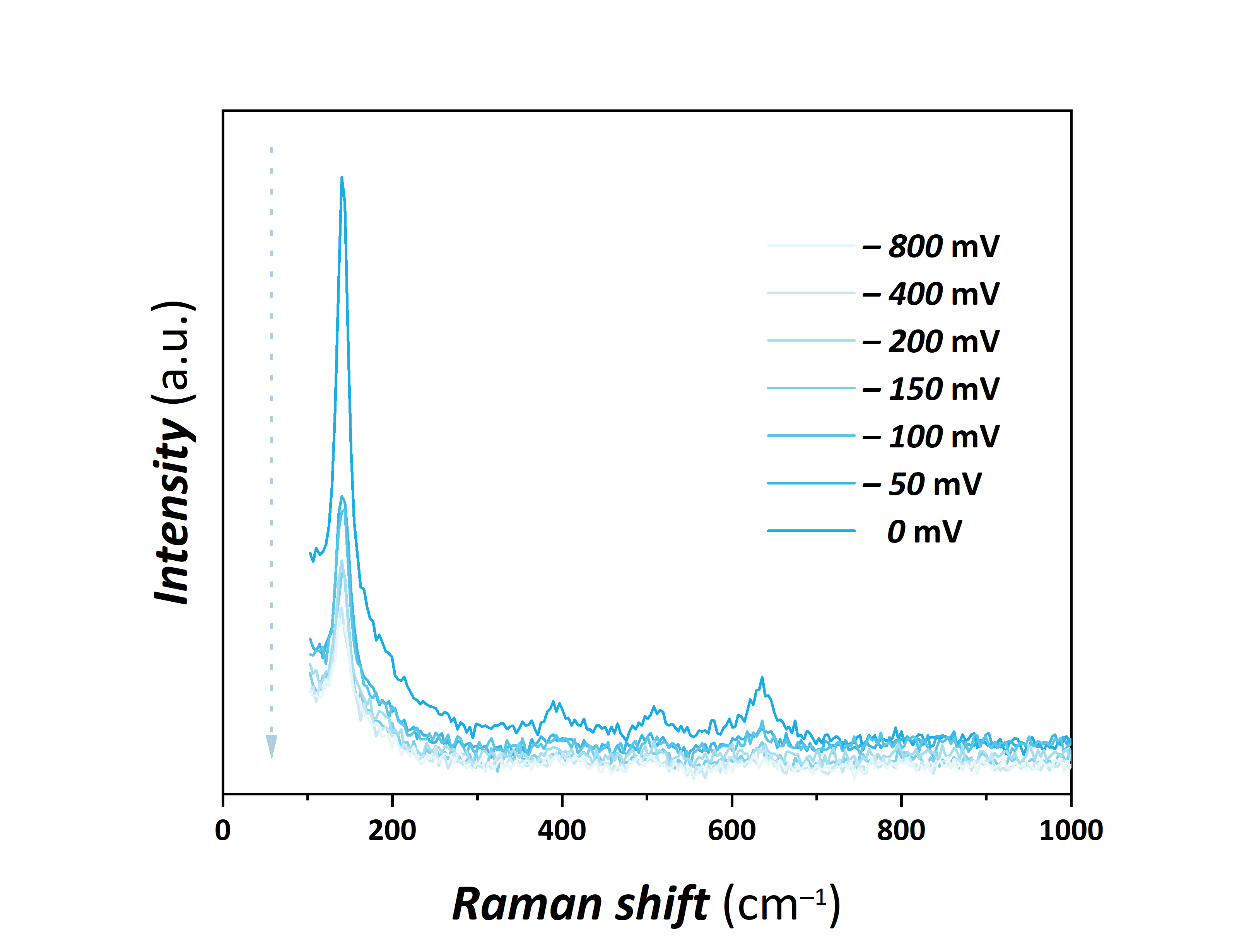


**Figure S23.** In-situ Raman spectra of the PtFeCoNiCu HEA/TiO_2_–NF synthesized by anodization at 120 V and electrodeposition at 1 V for 120 minutes in 1 M KOH with varied overpotentials, from 0 mV to –800 mV *vs* RHE at low range, from 0 to 1000 cm⁻^1^.


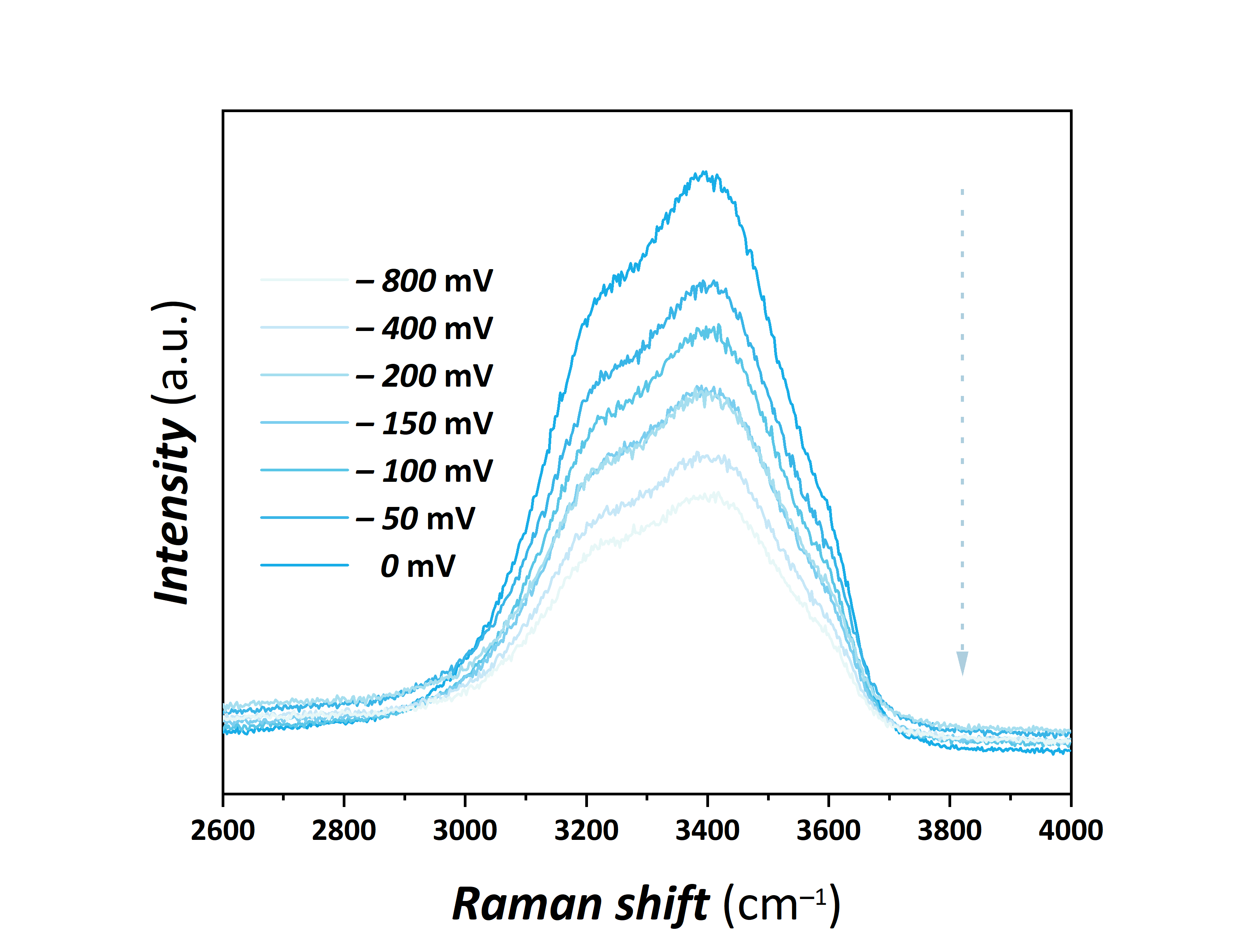


**Figure S24.** In-situ Raman spectra of the PtFeCoNiCu HEA/TiO_2_–NF synthesized by anodization at 120 V and electrodeposition at 1 V for 120 minutes in 1 M KOH with varied overpotentials, from 0 mV to –800 mV *vs* RHE at low range, from 2600 to 4000 cm⁻^1^.


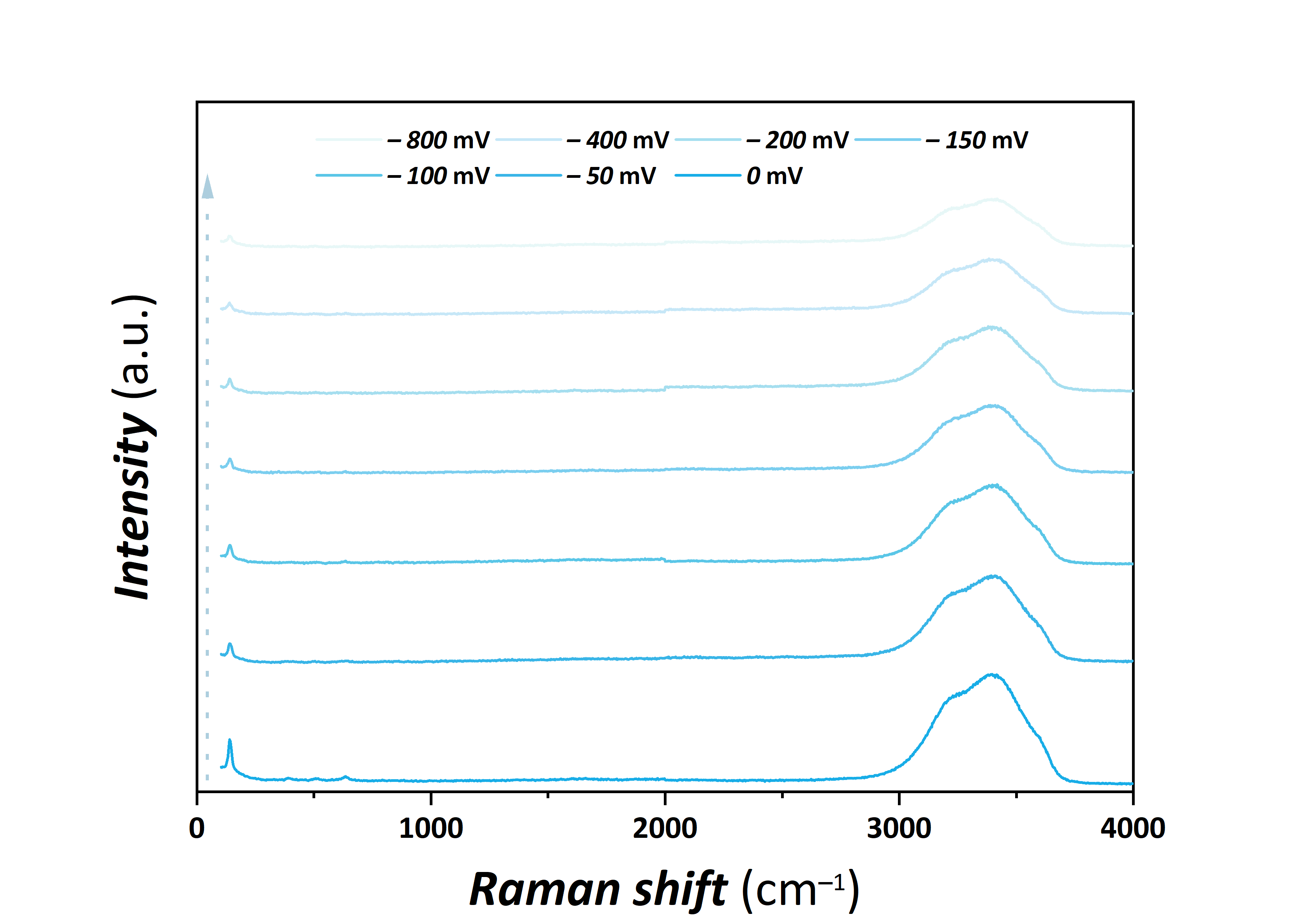


**Figure S25.** In-situ Raman spectra of the PtFeCoNiCu HEA/TiO_2_–NF synthesized by anodization at 120 V and electrodeposition at 1 V for 120 minutes in 1 M KOH with varied overpotentials, from 0 mV to –800 mV *vs* RHE at low range, from 0 to 4000 cm⁻^1^.


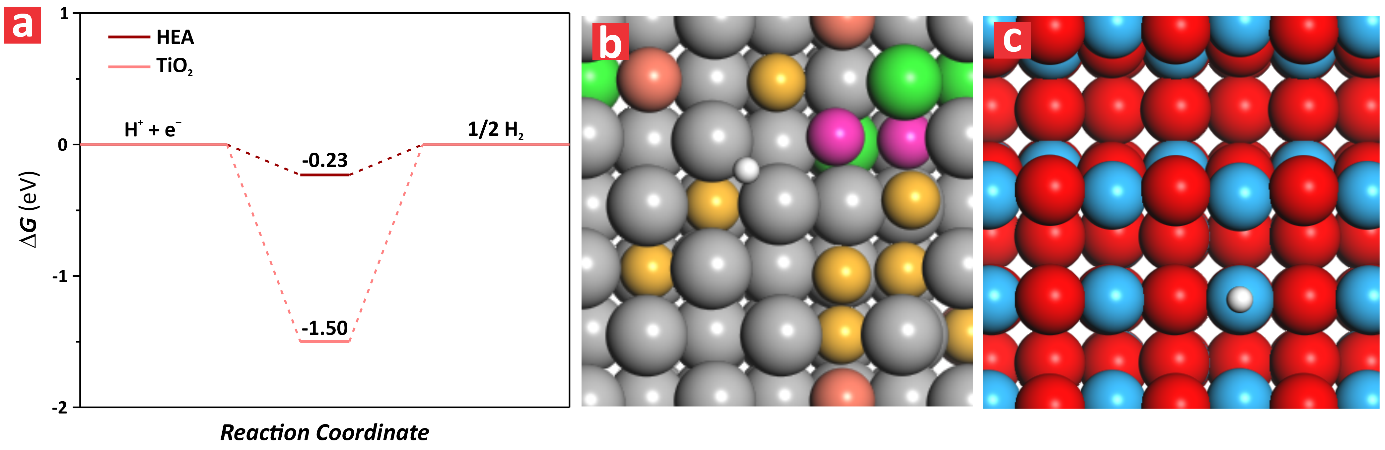


**Figure S26.** Reaction profile of one H atom binding and hydrogen evolution on PtFeCoNiCu HEA and anatase TiO_2_. (a) Gibbs free energy comparsion between PtFeCoNiCu HEA and anatase TiO_2_. (b) One H atom adsorption site on PtFeCoNiCu HEA. (c) One H atom adsorption site on anatase TiO_2._


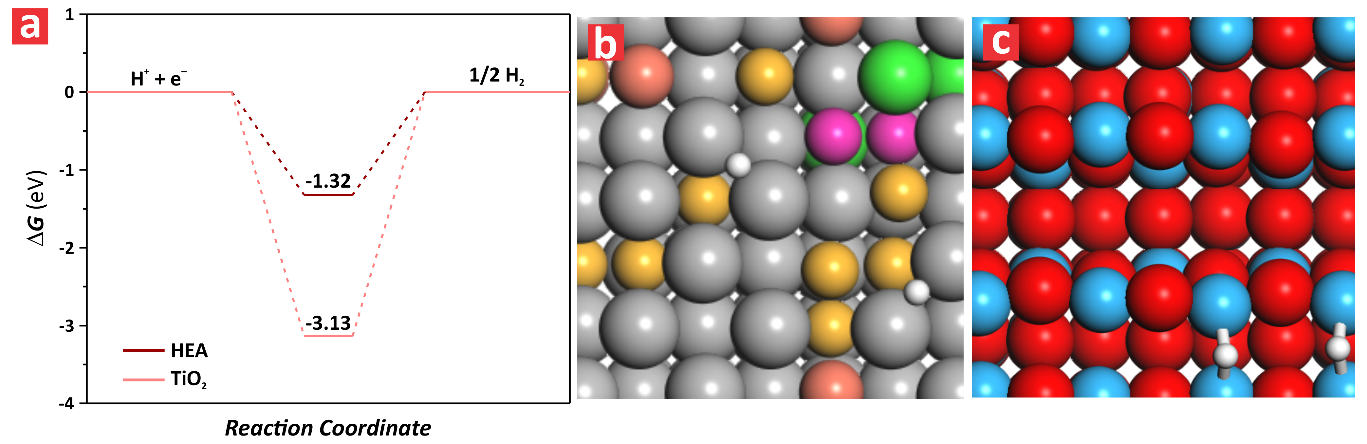


**Figure S27.** Reaction profile of two H atoms binding and hydrogen evolution on PtFeCoNiCu HEA and anatase TiO_2_. (a) Gibbs free energy comparsion between PtFeCoNiCu HEA and anatase TiO_2_. (b) Two H atoms adsorption site on PtFeCoNiCu HEA. (c) Two H atoms adsorption site on anatase TiO_2._


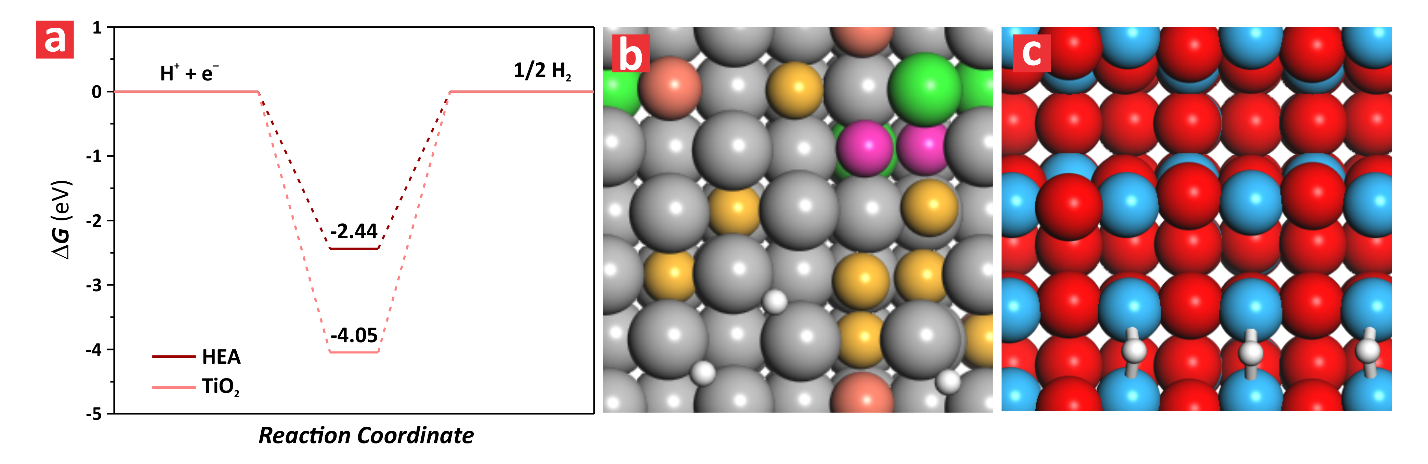


**Figure S28.** Reaction profile of three H atoms binding and hydrogen evolution on PtFeCoNiCu HEA and anatase TiO_2_. (a) Gibbs free energy comparsion between PtFeCoNiCu HEA and anatase TiO_2_. (b) Three H atoms adsorption site on PtFeCoNiCu HEA. (c) Three H atoms adsorption site on anatase TiO_2._


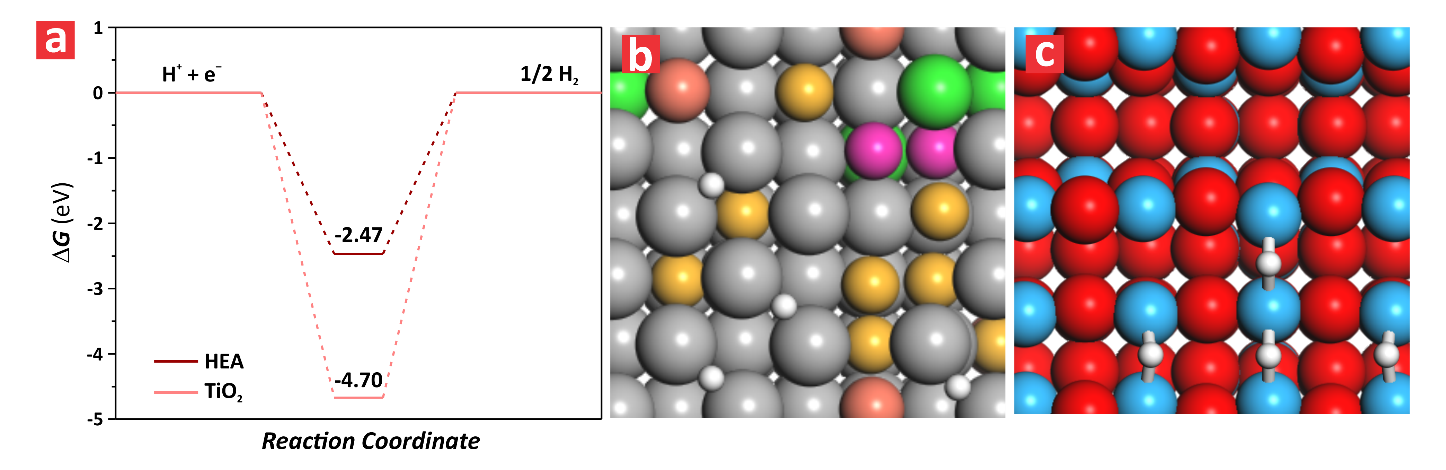


**Figure S29.** Reaction profile of four H atoms binding and hydrogen evolution on PtFeCoNiCu HEA and anatase TiO_2_. (a) Gibbs free energy comparsion between PtFeCoNiCu HEA and anatase TiO_2_. (b) four H atoms adsorption site on PtFeCoNiCu HEA. (c) four H atoms adsorption site on anatase TiO_2._


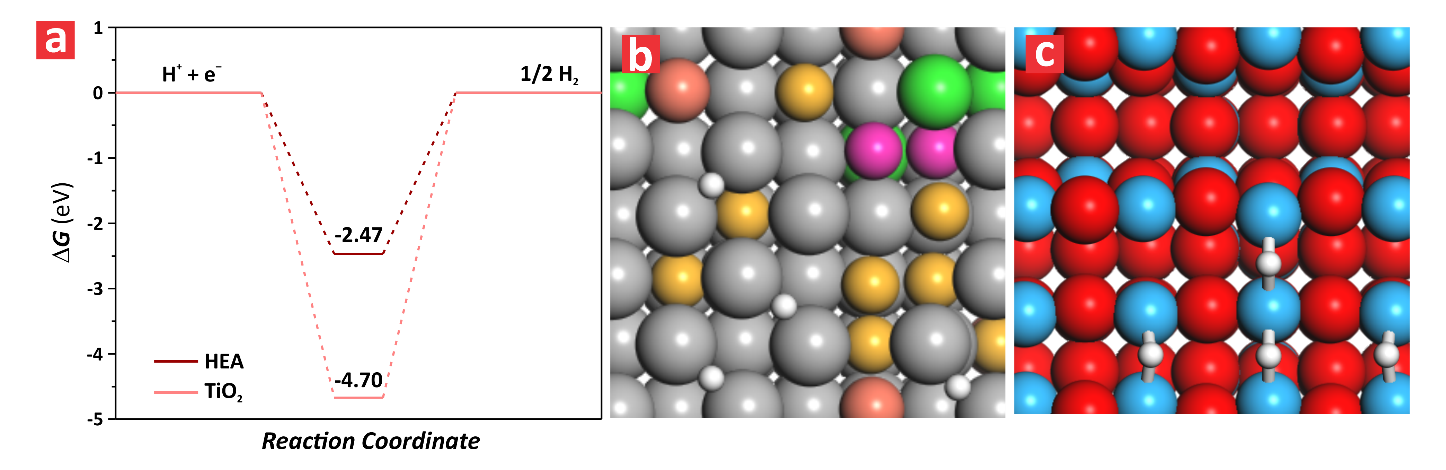


**Figure S30.** Reaction profile of five H atoms binding and hydrogen evolution on PtFeCoNiCu HEA and anatase TiO_2_. (a) Gibbs free energy comparsion between PtFeCoNiCu HEA and anatase TiO_2_. (b) five H atoms adsorption site on PtFeCoNiCu HEA. (c) five H atoms adsorption site on anatase TiO_2._


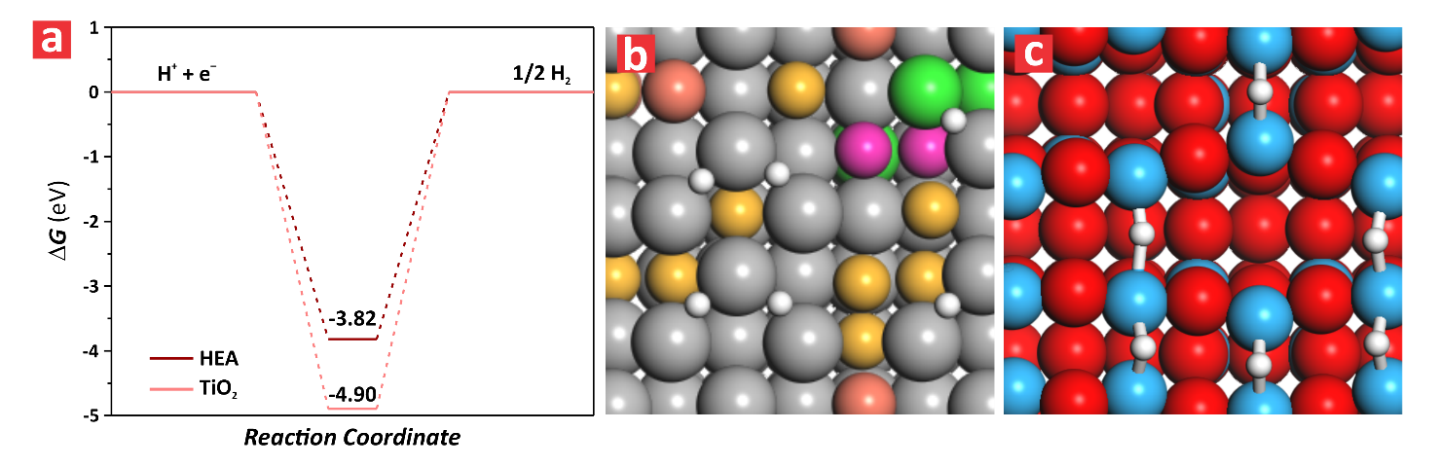


**Figure S31.** Reaction profile of six H atoms binding and hydrogen evolution on PtFeCoNiCu HEA and anatase TiO_2_. (a) Gibbs free energy comparsion between PtFeCoNiCu HEA and anatase TiO_2_. (b) six H atoms adsorption site on PtFeCoNiCu HEA. (c) six H atoms adsorption site on anatase TiO_2_.


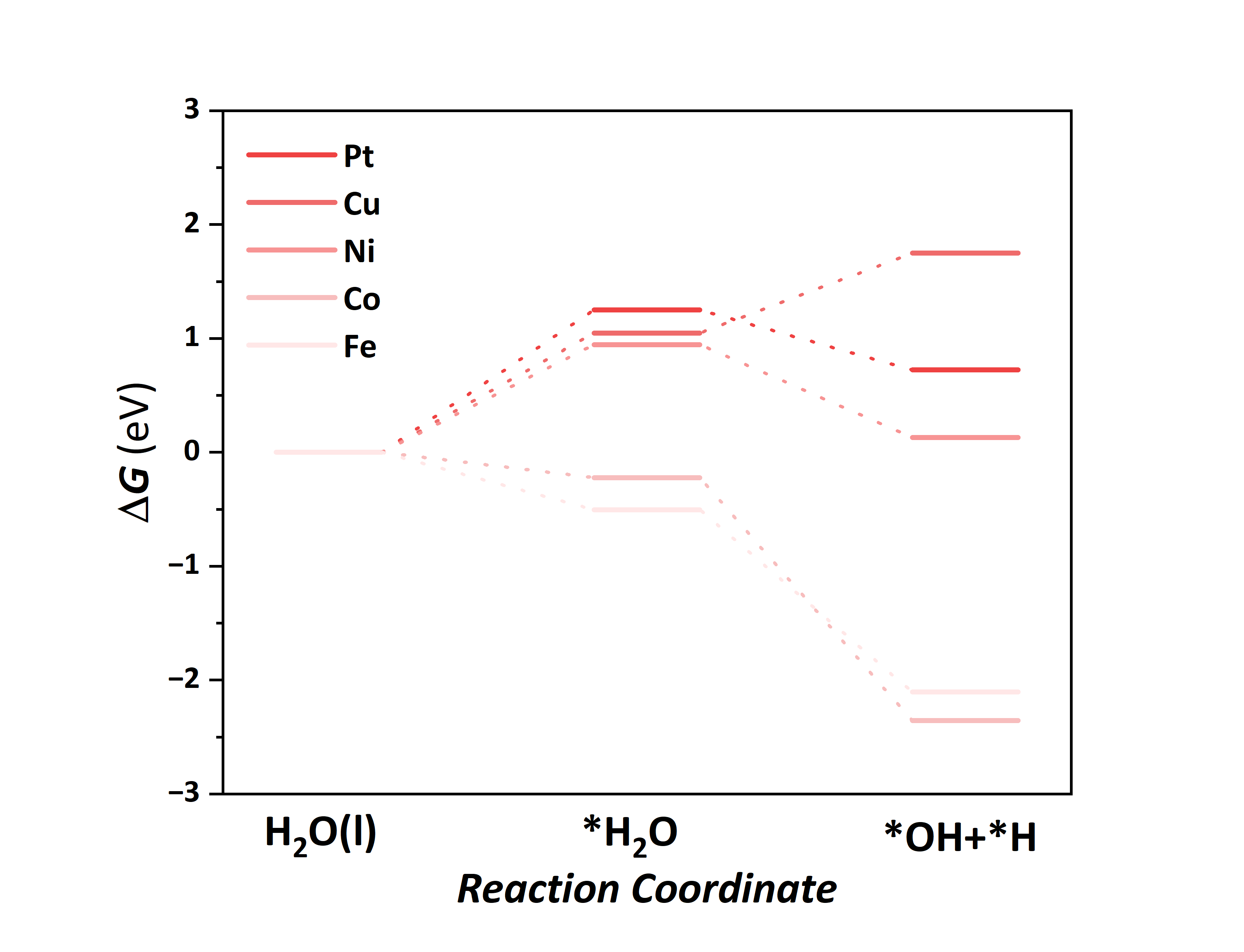


**Figure S32.** Gibbs free energy of H_2_O dissociation on the PtFeCoNiCu HEA.


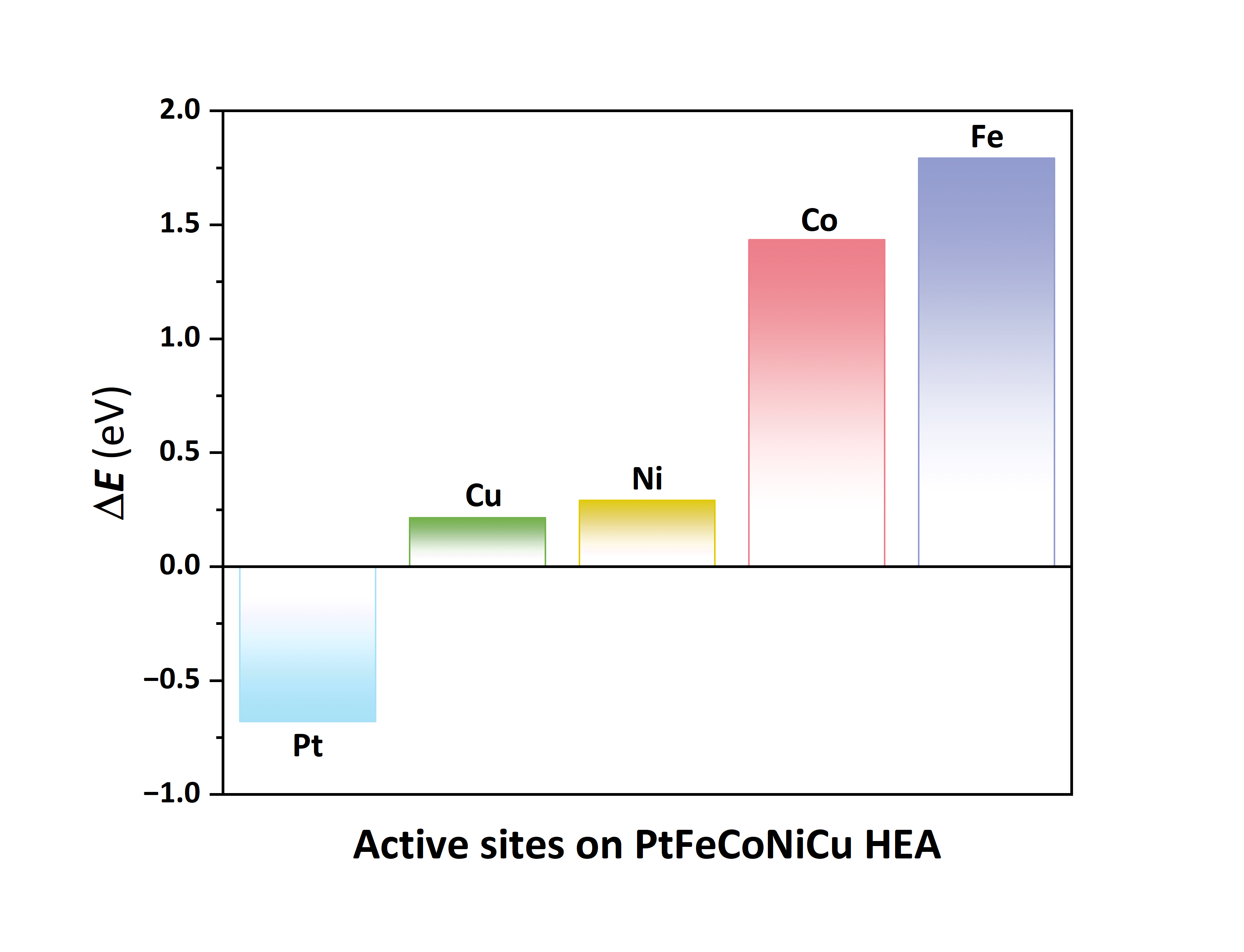


**Figure S33.** *H absorption energy of varied active sites on the PtFeCoNiCu HEA.


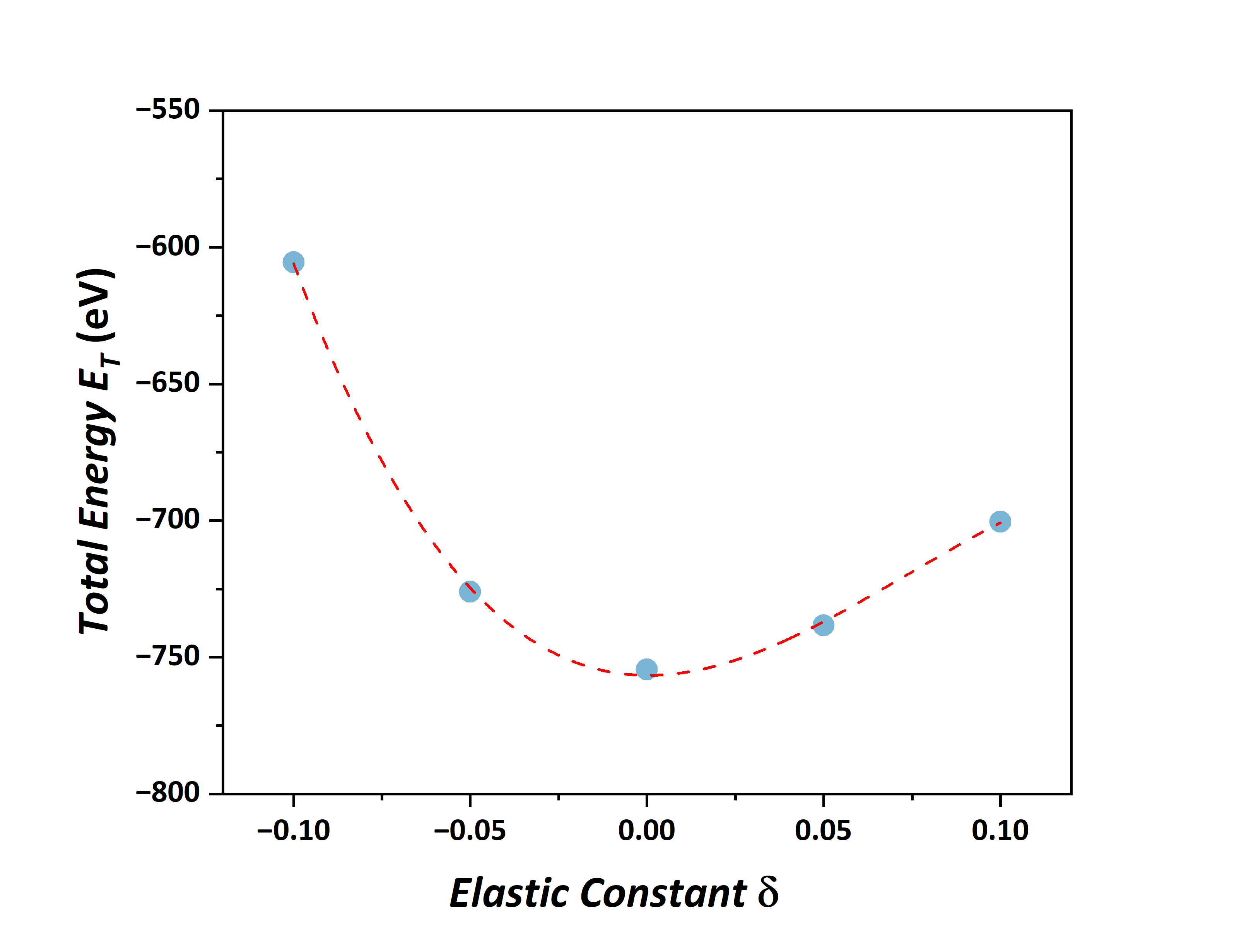


**Figure S34.** Total energy of the PtFeCoNiCu HEA structure under varied Elastic Constant by DFT.

**Table S1.** Gibbs free energy of hydrogen adsorption of PtFeCoNiCu HEA *via* increased reaction temperature from room temperature to 80 ^o^C and 1 H atom to 6 H atoms.

| **Temperature (^o^C)**  **H atoms** | **Gibbs free energy (G_ad_)** | | |
| --- | --- | --- | --- |
|  | RT | 50 | 80 |
| 1 | -0.2311 | -0.2327 | -0.2348 |
| 2 | -1.3207 | -1.3239 | -1.3282 |
| 3 | -2.4391 | -2.4432 | -2.4489 |
| 4 | -2.4668 | -2.4723 | -2.4800 |
| 5 | -3.0663 | -3.0729 | -3.0821 |
| 6 | -3.8194 | -3.8274 | -3.8386 |

**Table S2.** Gibbs free energy of hydrogen adsorption of anatase TiO_2_ *via* increased reaction temperature from room temperature to 80 ^o^C.

| **Temperature (^o^C)**  **H atoms** | **Gibbs free energy (G_ad_)** | | |
| --- | --- | --- | --- |
|  | RT | 50 | 80 |
| 1 | -1.4993 | -1.5001 | -1.5012 |
| 2 | -3.1328 | -3.1341 | -3.1360 |
| 3 | -4.0466 | -4.0486 | -4.0516 |
| 4 | -4.6697 | -4.6725 | -4.6766 |
| 5 | -4.9483 | -4.9518 | -4.9569 |
| 6 | -4.8950 | -4.8997 | -4.9067 |

**Table S3.** Comparison of HER performance at η_10_ between this work and recent studies on comparable systems.

| **Materials** | **Overpotential at η_10_ (mV)** | **Reference** |
| --- | --- | --- |
| **PtFeCoNiCu HEA/TiO_2_–NF** | 11 | **This work** |
| **PdPtRhIrCu HEA** | 15 | [2] |
| **PdFeCoNiCu HEA** | 18 | [3] |
| **IrPdPtRhRu HEA** | 17 | [4] |
| **FeCoNiCuPd HEA** | 29 | [5] |
| **PtPdRhIrNi HEA** | 55 | [6] |
| **PtFeNiCuMoRu HEA** | 20 | [7] |
| **MoO_x_-Rh metallene** | 15 | [8] |
| **Ir-NSG** | 18.5 | [9] |
| **SANi-Pt NWs** | 70 | [10] |
| **Pd@Ru NRs** | 37 | [11] |
| **RhSe_2_** | 81.6 | [12] |
| **Pt@PCM** | 139 | [13] |
| **NiFeMoCoCr HEA** | 172 | [14] |

**Table S4**. The structure parameters of anatase (001) and HEA (001).

|  | Anatase (001) | HEA (001) |
| --- | --- | --- |
| Cell length (a.b.c)(Å) | a = b = 11.35, c = 29.01 | a = b = 11.43, c = 24.52 |
| Cell angle (°) | α = β = γ = 90.00 | α = β = γ = 90.00 |
| Vacuum space (Å) | 15 | 15 |

The atom percentage of each metal in HEA is determined by the average values of XPS atomic percentage, EDS and ICP–MS, the amount of each metal is ordering as Pt (59.6%), Fe (21.0%), Cu (11.4%), Ni (5.6%) and Co (2.4%).

**Support Information References**

[1] S. Franz, H. Arab, G. L. Chiarello, M. Bestetti, E. Selli, *Adv. Energy Mater* **2020**, 10.

[2] Q. Mao, X. Mu, K. Deng, H. Yu, Z. Wang, Y. Xu, X. Li, L. Wang, H. Wang, *Adv. Funct. Mater* **2023**, 33.

[3] D. Zhang, Y. Shi, H. Zhao, W. Qi, X. Chen, T. Zhan, S. Li, B. Yang, M. Sun, J. Lai, B. Huang, L. Wang, *J. Mater. Chem. A* **2021**, 9, 889.

[4] D. Wu, K. Kusada, T. Yamamoto, T. Toriyama, S. Matsumura, I. Gueye, O. Seo, J. Kim, S. Hiroi, O. Sakata, S. Kawaguchi, Y. Kubota, H. Kitagawa, *Chem Sci* **2020**, 11, 12731.

[5] S. Wang, B. Xu, W. Huo, H. Feng, X. Zhou, F. Fang, Z. Xie, J. K. Shang, J. Jiang, *APPL CATAL B-ENVIRON ENERGY* **2022**, 313.

[6] Y. Yu, F. Xia, C. Wang, J. Wu, X. Fu, D. Ma, B. Lin, J. Wang, Q. Yue, Y. Kang, *Nano Res* **2022**, 15, 7868.

[7] Z. Chen, J. Wen, C. Wang, X. Kang, *Small* **2022**, 18, e2204255.

[8] J. Wu, J. Fan, X. Zhao, Y. Wang, D. Wang, H. Liu, L. Gu, Q. Zhang, L. Zheng, D. J. Singh, X. Cui, W. Zheng, *Angew. Chem* **2022**, 134.

[9] Q. Wang, C. Q. Xu, W. Liu, S. F. Hung, H. Bin Yang, J. Gao, W. Cai, H. M. Chen, J. Li, B. Liu, *Nat Commun* **2020**, 11, 4246.

[10] M. Li, K. Duanmu, C. Wan, T. Cheng, L. Zhang, S. Dai, W. Chen, Z. Zhao, P. Li, H. Fei, Y. Zhu, R. Yu, J. Luo, K. Zang, Z. Lin, M. Ding, J. Huang, H. Sun, J. Guo, X. Pan, W. A. Goddard, P. Sautet, Y. Huang, X. Duan, *Nat. Catal* **2019**, 2, 495.

[11] Y. Luo, X. Luo, G. Wu, Z. Li, G. Wang, B. Jiang, Y. Hu, T. Chao, H. Ju, J. Zhu, Z. Zhuang, Y. Wu, X. Hong, Y. Li, *ACS Appl Mater Interfaces* **2018**, 10, 34147.

[12] W. Zhong, B. Xiao, Z. Lin, Z. Wang, L. Huang, S. Shen, Q. Zhang, L. Gu, *Adv Mater* **2021**, 33, e2007894.

[13] H. Zhang, P. An, W. Zhou, B. Y. Guan, P. Zhang, J. Dong, X. W. D. Lou, *Sci Adv* **2018**, 4, eaao6657.

[14] G. Zhang, K. Ming, J. Kang, Q. Huang, Z. Zhang, X. Zheng, X. Bi, *Electrochim. Acta* **2018**, 279, 19.
